# Improved tag-switch method reveals that thioredoxin acts as depersulfidase and controls the intracellular levels of protein persulfidation†
†Electronic supplementary information (ESI) available. See DOI: 10.1039/c5sc04818d


**DOI:** 10.1039/c5sc04818d

**Published:** 2016-02-09

**Authors:** Rudolf Wedmann, Constantin Onderka, Shengwei Wei, István András Szijártó, Jan Lj. Miljkovic, Aleksandra Mitrovic, Mike Lange, Sergey Savitsky, Pramod Kumar Yadav, Roberta Torregrossa, Ellen G. Harrer, Thomas Harrer, Isao Ishii, Maik Gollasch, Mark E. Wood, Erwan Galardon, Ming Xian, Matthew Whiteman, Ruma Banerjee, Milos R. Filipovic

**Affiliations:** a Department of Chemistry and Pharmacy , Friedrich-Alexander University of Erlangen-Nuremberg , Erlangen , Germany . Email: milos.filipovic@ibgc.cnrs.fr; b Charité Campus Virchow , Nephrology/Intensive Care , Berlin , Germany; c Department of Chemistry , University of Belgrade , Belgrade , Serbia; d Department of Biological Chemistry , University of Michigan , Ann Arbor , USA; e University of Exeter Medical School , St. Luke's Campus , Exeter , UK; f Biosciences , College of Life and Environmental Sciences of Biosciences , University of Exeter , Streatham Campus , Exeter , Devon , UK; g Infectious Diseases Section , Department of Internal Medicine 3 , Universitätsklinikum Erlangen , Friedrich-Alexander-University, Erlangen-Nürnberg , Germany; h Department of Biochemistry , Graduate School of Pharmaceutical Sciences , Keio University , Tokyo , Japan; i UMR CNRS 8601 , Université Paris Descartes , Sorbonne Paris Cité , Paris , France; j Department of Chemistry , Washington State University , Pullman , USA; k Université de Bordeaux , IBGC , UMR 5095 , F-33077 Bordeaux , France; l CNRS , IBGC , UMR 5095 , F-33077 Bordeaux , France

## Abstract

Hydrogen sulfide (H_2_S) has emerged as a signalling molecule capable of regulating several important physiological functions such as blood pressure, neurotransmission and inflammation. The mechanisms behind these effects are still largely elusive and oxidative posttranslational modification of cysteine residues (protein persulfidation or *S*-sulfhydration) has been proposed as the main pathway for H_2_S-induced biological and pharmacological effects. As a signalling mechanism, persulfidation has to be controlled. Using an improved tag-switch assay for persulfide detection we show here that protein persulfide levels are controlled by the thioredoxin system. Recombinant thioredoxin showed an almost 10-fold higher reactivity towards cysteine persulfide than towards cystine and readily cleaved protein persulfides as well. This reaction resulted in H_2_S release suggesting that thioredoxin could be an important regulator of H_2_S levels from persulfide pools. Inhibition of the thioredoxin system caused an increase in intracellular persulfides, highlighting thioredoxin as a major protein depersulfidase that controls H_2_S signalling. Finally, using plasma from HIV-1 patients that have higher circulatory levels of thioredoxin, we could prove depersulfidase role *in vivo*.

## Abstract 2

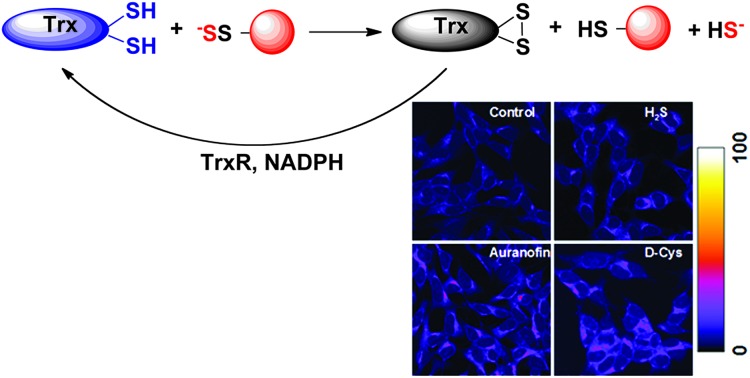
H_2_S signals *via* protein persulfidation. To be regulatory the modification will have to be reversible. Using a new method for persulfide detection, we discover this missing link and show that thioredoxin system acts as depersulfidase *in vivo*.

## Introduction

Oxidative posttranslational modifications (oxPTM) of protein cysteine residues are important for regulation of diverse cellular functions.^1^ From *S*-nitrosation^2^,^3^ and *S*-glutathionylation^4^,^5^ to *S*-sulfenylation,^1^,^6^ these modifications have been linked to signalling by reactive nitrogen and oxygen species. The recent emergence of a new signalling molecule, hydrogen sulfide (H_2_S), has provided insights into another oxPTM of cysteine, persulfidation (also called *S*-sulfhydration).^7^–^10^ Thus far, persulfidation of a handful of proteins has been demonstrated to be important for the regulation of blood pressure (*K*_ATP_ channel),^11^ cell senescence (Keap-1),^12^ and cell apoptosis (GAPDH and NF-κB).^7^,^13^ Additionally persulfidation of parkin increased its activity, suggesting that pharmacological H_2_S donors might be beneficial for the treatment of Parkinson's disease^14^ and possibly other neurodegenerative diseases.

Intracellular protein disulfide and *S*-glutathionylation levels are controlled by the thioredoxin (Trx) system.^15^–^17^ The enzymatic system, consisting of Trx, thioredoxin reductase (TrxR) and NADPH, represents the main disulfide reductase system in cells. Trx also regulates *S*-nitrosation.^18^,^19^ Numerous studies have shown that Trx acts as a denitrosylase and is also involved in selective *trans*-nitrosation (*i.e.* the transfer of an “NO^+^” moiety from an *S*-nitrosothiol to another cysteine residue).^20^–^22^ The latter is best illustrated by Trx-mediated caspase 3 *S*-nitrosation, which prevents apoptosis.^20^

To exert a regulatory function similar to that of phosphorylation/dephosphorylation or *S*-nitrosation/denitrosation, *S*-persulfidation levels must be enzymatically regulated.^9^,^10^ Due to the meta-stability of persulfides and their higher nucleophilicity (and therefore reactivity) compared to thiols, it is challenging to study their biochemistry. The lack of selective tools for labelling persulfides additionally complicates these studies.^23^,^24^ We have recently developed a tag-switch method for selective persulfide labelling^25^,^26^ and assessed alternative mechanisms for persulfide formation, suggesting that persulfides could be formed from sulfenylated cysteine residues.^27^ Some evidence already exists to suggest that Trx can cleave persulfide from the active site of mercaptopyruvate sulfur transferase (MST) leading to H_2_S release.^28^,^29^ To asses the global role of Trx system on protein persulfidation, we herein developed an improved tag-switch assay and showed that the Trx system reacts approximately an order of magnitude faster with persulfides than their disulfide analogs, and is the major regulator of protein persulfide levels in the cells.

## Results and discussion

**Fig. 1:**
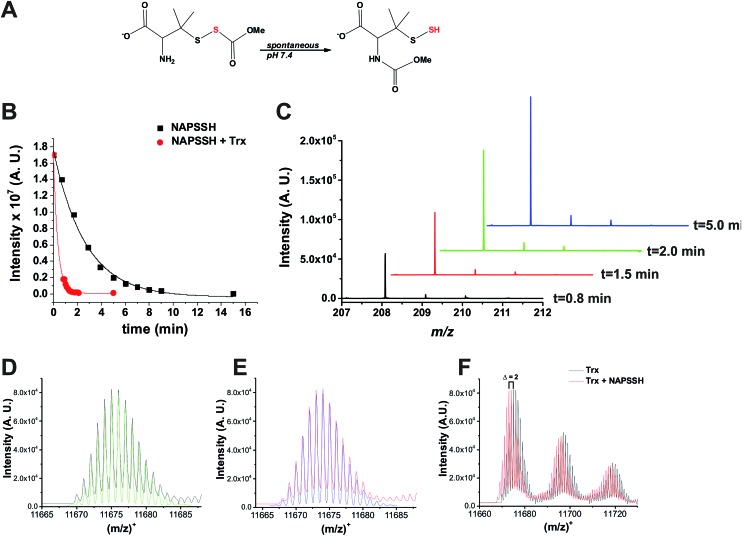
Trx reacts with LMW persulfide NAP-SSH. (A) NAP-SSH undergoes spontaneous re-arrangement in buffer to give persulfide analogs. (B) Kinetics of 50 μM NAP-SSH decay followed by MS. Disappearance of the NAP-SSH parent ion (*m*/*z* 240.0347; calculated 240.0359), in the absence (black squares) and presence of 50 μM Trx (red circles), was plotted over time. (C) Time resolved MS spectra of *m*/*z* 208.0623 (calculated 208.0638) peak, which corresponds to NAPSH, indicate that Trx cleaves NAP-SSH to form a thiol and release HS^–^. See Fig. S1 in ESI.† (D) Deconvoluted mass spectrum of [Trx_red_ + H]^+^ (black) and simulated isotopic distribution for the fully reduced Trx (C_528_H_838_N_132_O_159_S_3_; green). See Fig. S2 in ESI.† (E) Deconvoluted mass spectrum of Trx mixed with NAP-SSH (red) and simulated isotopic distribution of fully oxidized Trx (C_528_H_836_N_132_O_159_S_3_; blue). See Fig. S2 in ESI.† (F) Overlay of the starting Trx spectrum and the spectrum obtained after 2 min of incubation with NAP-SSH clearly indicates *m*/*z* 2 leftward shift, indicative of the loss of 2H atoms.

To assess the potential role of Trx in persulfide reduction, we first used the low molecular weight (LMW) persulfide analogue of *S*-nitrosopenicillamine (NAP-SSH)^30^ (Fig. 1A). NAP-SSH spontaneously decomposes when added in the buffer, as some of us demonstrated previously.^30^ The reaction was monitored by time-resolved mass spectrometry. NAP-SSH is metastable and its decay was monitored by the disappearance of the parent ion peak (at *m*/*z* 240.0347; calculated 240.0359 Fig. 1B and S1 in ESI†), and the appearance of peaks with *m*/*z* 208.0623 (calculated 208.0638) and 225.0883 (calculated 225.0904) corresponding to [NAPSH + H]^+^ and [NAPSH + NH_4_]^+^, respectively (Fig. 1C and S1 in ESI†). The half-time for the spontaneous decay of 50 μM NAP-SSH in ammonium carbonate buffer pH 7.38 was estimated to be 2.7 ± 0.3 min and this is in good agreement with previous chemical studies on its stability.^30^ The presence of equimolar Trx (*E. coli*) increased the decay of NAP-SSH ∼10-fold (*t*_1/2_ = 0.3 ± 0.1 min). The mass spectrum of Trx after 2 min incubation with a 2-fold excess of NAP-SSH showed the complete oxidation of Trx. The complete mass spectra of reduced Trx and Trx treated with NAP-SSH are shown in Fig. S2 in ESI,† and the isotopic distribution of the observed and simulated reduced [Trx_red_ + H]^+^ and oxidized [Trx_ox_ + H]^+^ are shown in Fig. 1D and E, respectively. Complete oxidation is demonstrated by the leftward *m*/*z* 2 shift (Fig. 1F). Two reaction mechanisms are possible to explain the reaction: (i) transfer of the sulfane sulfur from NAP-SSH to the nucleophilic cysteine of Trx leads to the transient formation of Trx-SSH followed by the displacement of the sulfide anion by the resolving cysteine and formation of a disulfide bond, or (ii) a nucleophilic attack of one of the Trx cysteines to the sulfane sulfur of NAP-SSH with immediate elimination of H_2_S and formation of Trx-NAPSH disulfide complex, followed by the displacement of NAPSH by the resolving cysteine and formation of a disulfide bond. Either mechanism could result in H_2_S generation. Therefore we examined H_2_S production from 100 μM NAP-SSH treated with 10 μM Trx using an H_2_S-selective electrode. While a H_2_S-specific current was not detected with either Trx or NAP-SSH alone (the latter decomposes spontaneously to give a mixture of tri, tetra and pentasulfides of NAPSH),^30^ the addition of NAP-SSH to Trx-containing buffer induced an immediate electrode response (Fig. S3 in ESI†) indicative of H_2_S formation.

**Fig. 2:**
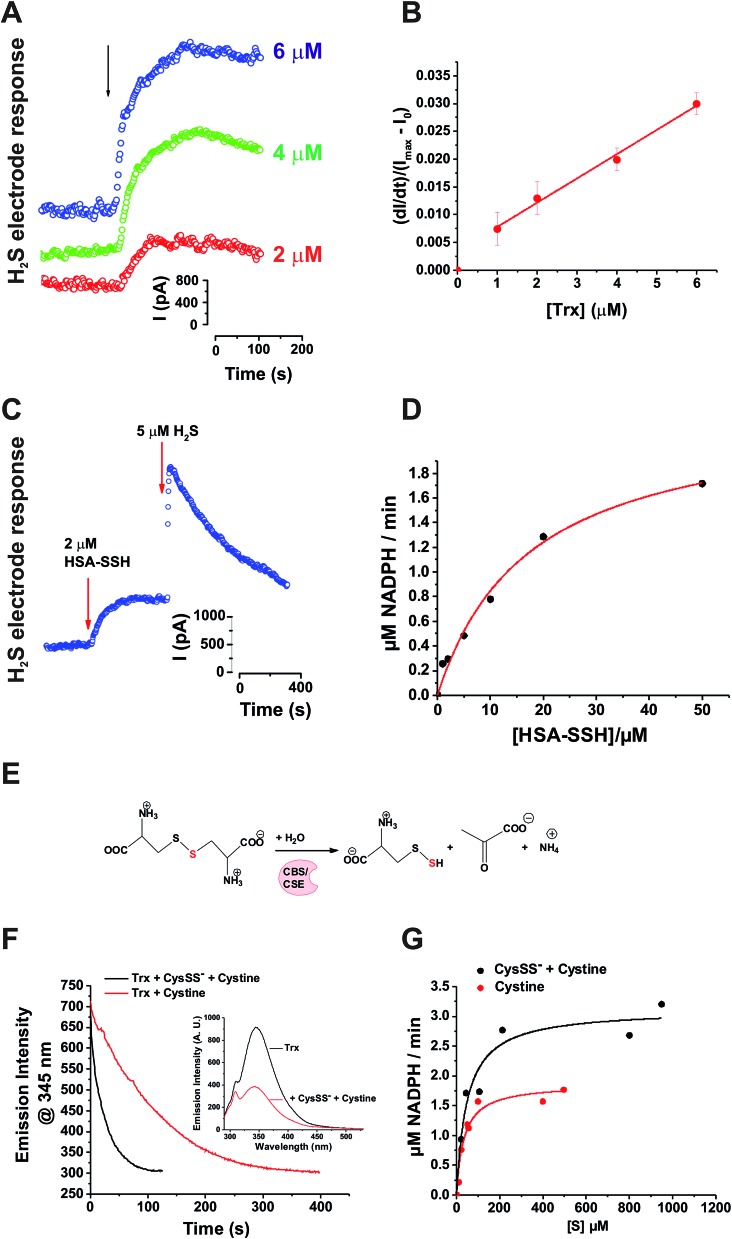
Trx system cleaves cysteine and protein persulfides to form H_2_S. (A) H_2_S release from 20 μM HSA-SSH upon addition of different concentrations of Trx (*E. coli*), measured amperometrically by the H_2_S sensitive electrode. (B) Plot of the initial rates *vs.* the concentration of Trx. (C) Combined with TrxR (rat) and NAPDH, Trx (human) efficiently release all sulfur trapped in HSA-SSH as H_2_S. Na_2_S was injected as internal standard for the comparison. (D) Kinetic analysis of the reaction was performed by measuring the rates of NADPH oxidation varying the concentration of HSA-SSH and keeping the concentrations of other parameters constant: 1 μM Trx (human), 0.01 μM TrxR (rat) and 250 μM NADPH. Experiments were performed in triplicates. (E) Schematic overview of the reaction used for the generation of the CysSS^–^/CysSSCys mixture. (F) Kinetics of Trx (*E. coli*) oxidation (1 μM) with 10 μM CysSSCys (black line) or CysSS^–^/CysSSCys mixture (red line), followed by tryptophan fluorescence (*λ*_ex_ 280 nm) changes. Inset: Emission spectra before and after the reaction of 1 μM Trx with 50 μM CysSS^–^/CysSSCys mixture. See Fig. S5 in ESI.† (G) Kinetic analysis of the reaction of CysSSCys (black line) or CysSS^–^/CysSSCys mixture (red line) with 1 μM Trx (human), 0.01 μM TrxR (rat) and 250 μM NADPH.

Although Trx showed activity towards LMW persulfides, protein-bound persulfides are expected to represent a larger sulfane sulfur pool. Therefore, we tested the reaction of Trx with protein persulfides. We recently reported preparation of human serum albumin persulfide (HSA-SSH) and described its reactivity.^27^ Addition of Trx (*E. coli*) to a solution containing 20 μM HSA-SSH led to a concentration-dependent increase in the initial rate of H_2_S production, as monitored by an H_2_S-sensitive electrode (Fig. 2A), confirming our hypothesis that Trx reduces protein persulfides in addition to LMW persulfides. Using an initial rate approach, a second order rate constant for the reaction of Trx with HSA-SSH was estimated to be 4.1 ± 0.8 × 10^3^ M^–1^ s^–1^ (Fig. 2B). MS analysis of the reaction mixture containing 20 μM Trx and 40 μM HSA-SSH revealed complete oxidation of Trx (Fig. S4 in ESI†) as observed with NAP-SSH (Fig. 1F).

In cells, the disulfide bond in the active site of Trx is reduced by TrxR, which uses NADPH as an electron source. The catalytic cycling of Trx between the oxidized and reduced states was observed when 2 μM HSA-SSH was added to a solution containing substoichiometric Trx (0.5 μM) in the presence of TrxR and NADPH (0.007 and 250 μM), which led to a 95 ± 3% recovery of H_2_S from HSA-SSH (Fig. 2C).

We next assessed the kinetic parameters for the depersulfidation of HSA-SSH by the Trx/TrxR system. The dependence of the initial rate of NADPH consumption on the concentration of HSA-SSH exhibited Michaelis–Menten-like kinetic behaviour (Fig. 2D) yielding the apparent *K*_m_ and *V*_max_ parameters shown in Table 1. The apparent *k*_cat_*/K*_m_ value is similar to that reported for insulin reduction by Trx,^31^ which is one of the best substrates for the enzyme, indicating that the Trx/TrxR system can also readily cleave protein persulfides.

**Table 1:** Kinetic parameters for the Trx/TrxR/NADPH-catalysed depersulfidase reaction. 1 μM Trx, 0.01 μM TrxR and 250 μM NADPH were used for the reaction. Kinetic parameters for CysSS^–^ from the mixture of CysSS^–^ and CysSSCys were derived using the equation given by Pocklington and Jeffery, 1969 (ref. 33)

Substrate	*K*_m_ (μM)	*V*_max_ (μM min^–1^)	*k*_cat_/*K*_m_ × 10^5^^*a*^ (M^–1^ min^–1^)	*k*_cat_/*K*_m_ × 10^7^^*b*^ (M^–1^ min^–1^)	*k*_1_ x 10^3^^*c*^ (M^–1^ min^–1^)
HSA-SSH	17 ± 3	2.4 ± 0.2	1.4	1.4	246 ± 48
CysSSCys	35 ± 8	1.9 ± 0.1	0.5	0.5	30 ± 6
CysSSH	13 ± 5	5.1 ± 0.7	3.9	3.9	270 ± 6

^*a*^Calculated based on [Trx] (human).

^*b*^Calculated based on [TrxR] (rat).

^*c*^ *k*_1_ refers to the rate constant for the reaction between Trx and the substrate in the absence of TrxR/NADPH.

A recent study indicated the existence of high levels of intracellular and circulating LMW cysteine and glutathione persulfides and suggested that uncatalyzed *trans*-persulfidation reactions between LMW persulfides and cysteine residues lead to protein persulfidation.^32^ This study also showed that the H_2_S-producing enzymes, γ-cystathionase (CSE) and cystathionine β-synthase (CBS), produce large amounts of cysteine persulfide (Cys-SSH/Cys-SS^–^) from cystine (CysSSCys) (Fig. 2E). We therefore tested the reactivity of Trx towards Cys-SSH. Recombinant human CSE was incubated with CysSSCys at 37 °C for 15 min before separating the reaction mixture from the protein. Approximately 50% of the substrate was converted to product during this time as assessed using Ellman's reagent. Transpersulfidation kinetics was initially monitored by spectrofluorimetry^31^ measuring the tryptophan fluorescence changes caused by Trx oxidation (Fig. 2F). The dependence of the *k*_obs_ of the fluorescence on the concentration of either CySSCys or the CySSCys/CysSS^–^ mixture, yielded rate constants for the oxidation of Trx by CySSCys and CysSS^–^ of 5 ± 1 × 10^2^ M^–1^ s^–1^ and 4.5 ± 0.1 × 10^3^ M^–1^ s^–1^, respectively (Table 1, Fig. S5 in ESI†). When the Trx/TrxR/NADPH system was used, Michaelis–Menten-like kinetic behaviour was observed (Fig. 2G) with the CysSS^–^/CysSSCys mixture yielding ∼2-fold higher *V*_max_ than CySSCys alone. Knowing the actual concentrations of both CysSS^–^ and CysSSCys in the reaction mixture, we used a competitive two-substrate equation^33^ to estimate the kinetic parameters for CysSSH (Table 1). This analysis revealed that the *k*_cat_/*K*_*m*_ for CySS^–^ (3.9 × 10^5^ M^–1^ min^–1^) is almost an order of magnitude greater than for CysSSCys (0.5 × 10^5^ M^–1^ min^–1^). It is important to note that CysSSH, like NAP-SSH, is intrinsically unstable in solution and it equilibrates to give a mixture of cysteine polysulfides. Therefore, the kinetic values reported here for CysSSH are likely to be an underestimation.

**Fig. 3:**
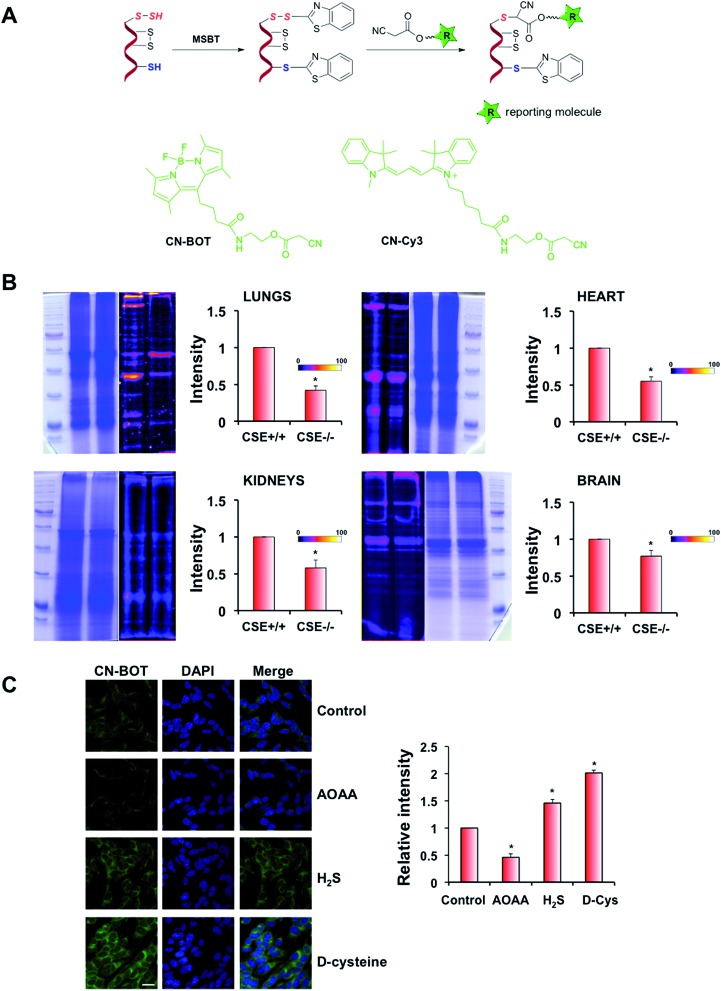
Improved tag-switch assay labels persulfides in cell lysates and fixed cells. (A) Original tag-switch assay with chemical structures of green fluorescence cyanoacetic acid derivative, CN-BOT and cyanoacetic acid derivative, CN-Cy3. (B) Validation of CN-Cy3 labelling of protein persulfides in tissue extracts from CSE^+/+^ and CSE^–/–^ mice (*n* = 3, *p < 0.01). Protein ladder in decreasing order: 245, 180, 135, 100, 75, 63, 48, 35, 25, and 20 kDa. (C) Validation of CN-BOT labelling of protein persulfidation in fixed cells. A decrease of intracellular persulfidation in SH-SY5Y neuroblastoma cells could be observed after 1 h incubation with CBS inhibitor AOAA (2 mM). Incubation with 100 μM Na_2_S or 2 mM d-cysteine (1 h) leads to the increase of protein persulfidation. Semi-quantification of *n* = 100 cells was performed using ImageJ software. Data represent mean ± S.E.M., *p < 0.01.

One of the main problems for studying cellular persulfidation is the lack of selective tools for detecting protein persulfides. Several methods have been described to date including the tag-switch assay.^7^,^13^,^25^ This latter method is based on the strategy of forming a mixed disulfide using methylsulfonyl benzothiazole (MSBT), an aromatic thiol-blocking reagent.^25^ The resulting mixed disulfide is more reactive towards the biotin-tagged cyanoacetic acid nucleophile than towards cysteine disulfides present in proteins (Fig. 3A). The method shows selectivity for persulfides without detectable cross-reactivity with other post-translational modifications of cysteine that have been tested.^25^,^26^

The original assay used a biotinylated cyanoacetic acid tag, which requires Western-blot transfer and streptavidin or antibodies for visualization.^25^,^26^ To increase sensitivity, we synthesized two new cyanoacetic acid derivatives with the fluorescent BODIPY moiety (CN-BOT) or the Cy3-dye (CN-Cy3) (Fig. 3A, Scheme S1–S3 in ESI,† for details see Experimental section in ESI†). Both new tags labelled HSA-SSH, yielding fluorescent products (Fig. S6 in ESI†). Preliminary studies using cell lysates showed that the CN-Cy3 tag gave better resolution of labelled bands and higher fluorescence intensity than the CN-BOT tag. However, when fixed cells were used, CN-Cy3 proved difficult to wash out (visible colouring of the washing solution was noticed even 1 week after washing). Therefore CN-BOT was used for labelling cells for microscopy and CN-Cy3 for the labelling in cell lysates.

The increase in sensitivity could be best illustrated by comparing CN-Biotin with CN-Cy3 labelling, as shown in Fig. S7 in ESI.† We used human erythrocytes and homogenates of *Drosophila melanogaster* heads. Although persulfidation was detected by Western blot in the samples labelled by CN-Biotin, stronger signals as well as higher numbers of protein bands were detected in samples labelled by CN-Cy3, demonstrating significant improvement of the sensitivity (Fig. S7 in ESI†).

Brain, heart, kidney and lung tissue extracts from 3C57BL/6 CSE^+/+^ and CSE^–/–^ mice^34^ were also labelled with CN-Cy3. Lower signal intensity indicative of lower total protein persulfidation was observed in all tissue extracts from CSE^–/–^ mice confirming the method's selectivity, with the effects being the strongest in lungs and heart and only modest in brain, which is consistent with the predominant roles of CBS and mercaptropyruvate sulfur transferase (MST) in brain (Fig. 3B).

Protein persulfidation in fixed cells was visualized by confocal microscopy using the tag-switch assay and the CN-BOT reagent. As a negative control, cells were only treated with CN-BOT without the initial MSBT blocking step, and as a positive control, cells were pre-treated with H_2_S (1 h with 100 μM Na_2_S). The signal intensity was barely detectable when CN-BOT was used alone, and 20-fold greater laser intensity was needed to observe a signal (Fig. S8A and B in ESI†). Treatment with Na_2_S led to a substantial increase in fluorescence intensity. Further validation of the CN-BOT tag-switch assay was obtained from experiments in which the CSE inhibitor propargylglycine (PG)^25^ and CBS inhibitor aminooxyacetic acid (AOAA) were used.^35^ Both inhibitors decreased the signal intensity in bovine arterial endothelial (BAE) and SH-SY5Y neuroblastoma cells (albeit not completely), respectively (Fig. 3C and S8C in ESI†), while incubation with Na_2_S or d-cysteine (an alternative substrate for MST-catalyzed H_2_S generation)^36^ increased the fluorescence signals. These microscopy results were in good agreement with persulfide analysis in cell lysates (Fig. S9 in ESI†).

**Fig. 4:**
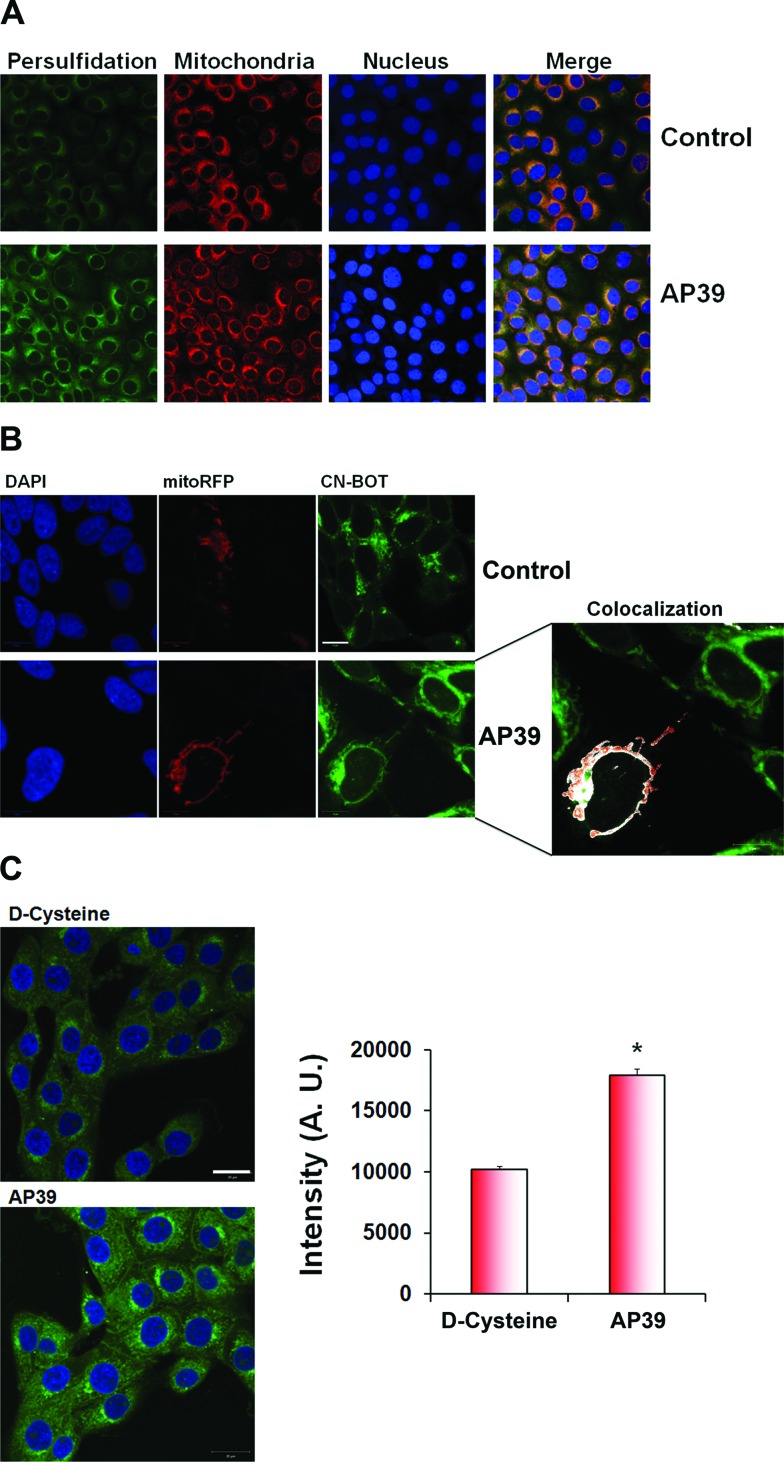
Persulfidation co-localizes with mitochondria. (A) Co-localization studies of intracellular persulfidation. BAECs were transfected with RFP fused to the leader sequence of E1 alpha pyruvate dehydrogenase and incubated with mitochondria-targeted H_2_S donor, AP39. (B) SH-SY5Y neuroblastoma cells were transfected with CellLight® Mitochondria-RFP, BacMam 2.0 to visualize mitochondria and the cells were stained with improved tag-switch assay for persulfidation. Analysis with ImageJ (version 1.45R) revealed that significant co-localization was evident, which was visually represented as white pixels using the co-localization highlighter. Mitochondria-targeted H_2_S donor, AP39, lead to a strong increase in mitochondrial persulfidation. Scale bar 10 μm. (C) 100 nM AP39 is much more powerful inducer of persulfidation in the cells than 2 mM d-cysteine. Scale bar 20 μm.

To further validate the microscopy results, we transfected mitochondria with RFP fused to the leader sequence of E1 alpha pyruvate dehydrogenase (CellLight®, Thermo Fischer Scientific) and treated the cells with AP39, a novel mitochondria targeted H_2_S donor.^37^–^39^ Based on co-localization studies, a significant portion of persulfidation appeared to be localized in mitochondria in the presence or absence of 100 nM AP39 (Fig. 4A and B). AP39 induced a strong increase in intracellular persulfidation, ∼2-fold higher than observed with 1 mM d-cysteine (Fig. 4C), suggesting that this compound can be a powerful pharmacological tool for modulating mitochondrial and intracellular H_2_S-mediated persulfidation.

**Fig. 5:**
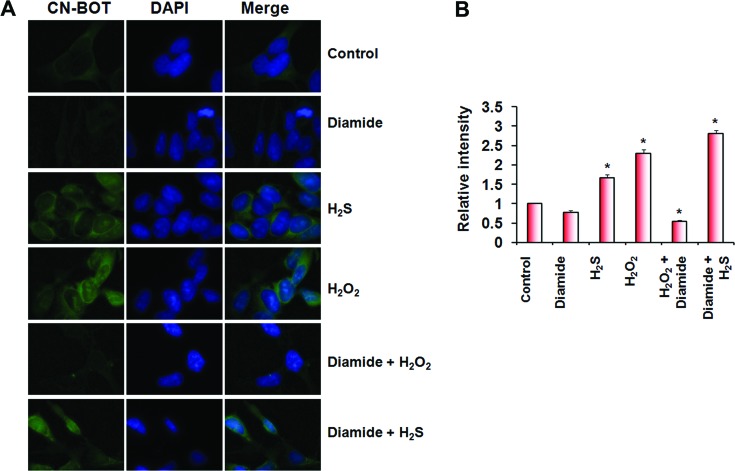
Reaction of H_2_S with sulfenic acids and not with disulfides could be a mechanism for intracellular persulfidation. (A and B) Incubation of SH-SY5Y neuroblastoma cells with 0.5 mM diamide (30 min) does not increase intracellular persulfidation, while the incubation with H_2_O_2_ (100 μM, 30 min) leads to a higher intracellular persulfide content (A). Treatment with 100 μM Na_2_S (1 h) was used as a positive control. At least 5 images were recorded from each experiment performed in triplicate. (B) Semi-quantification of 40 cells was performed using ImageJ software. Data represent mean ± S.E.M, *p < 0.01.

H_2_S cannot react directly with cysteine thiols, and a plausible mechanism for persulfide formation is the reaction of H_2_S with sulfenic acids or with disulfides.^1^,^10^ We recently characterized the kinetics of these two reactions although *a priori*, the reaction of H_2_S with disulfides is less likely to be important in the cellular milieu, which is reducing.^27^ Cells treated with H_2_O_2_ showed increased persulfidation, relative to control cells, and that could have been formed *via* either mechanism (Fig. 5A and B). To distinguish between these mechanistic possibilities, we treated cells with the thiol oxidants, diamide (which induces disulfide bond formation) and H_2_O_2_ (which induces sulfenic acids in addition to disulfides). While H_2_O_2_-treatment of SH-SY5Y cells resulted in a significant increase in fluorescence intensity indicative of protein persulfidation, treatment with diamide led to a slight decrease in intensity compared to untreated controls (Fig. 5A and B), confirming that H_2_S-induced persulfide formation would be a consequence of its reaction with sulfenic acids and not with protein disulfides.

**Fig. 6:**
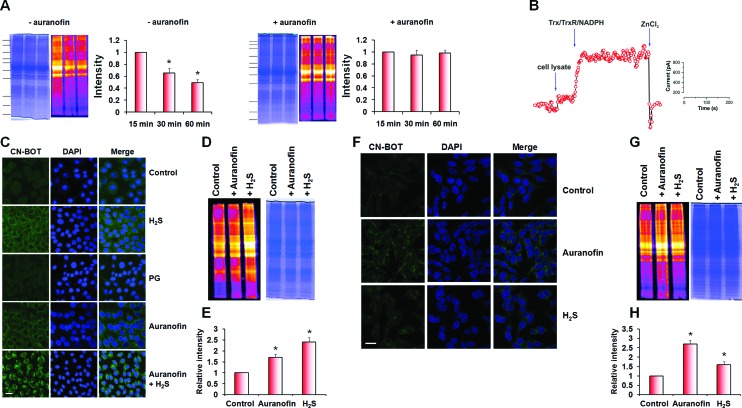
Trx system is the main regulator of intracellular persulfide levels. (A) Due to the activity of Trx system in the cell lysates obtained under native conditions (HEN buffer, pH 7.4, 1% protease inhibitors, 0 °C), levels of persulfides detected by improved tag-switch assay decrease with the time of protein extraction (gel on the left). This can be completely prevented by the addition of 2 μM auranofin into the lysis buffer (gel on the right). Data are presented as mean ± S.D (*n* = 3), *p < 0.01. Protein ladder in decreasing order: 200 kDa, 150 kDa, 120 KDa, 100 kDa, 70 kDa, 60 kDa, 50 kDa, 40 kDa, 30 kDa, 25 kDa and 20 kDa. (B) Representative amperometric measurement of H_2_S release from BAEC lysate (1 mg mL^–1^ protein). Cell lysate was added first (first arrow), followed by the addition of a mixture containing 1 μM Trx, 0.01 μM TrxR and 250 μM NADPH (second arrow). 100 μM ZnCl_2_ was added in the end to prove that the response is indeed from H_2_S. (C–E) Inhibition of Trx system with 2 μM auranofin for 1 h, leads to an increase of intracellular persulfidation in BAE cells in a similar way like the treatment with 100 μM Na_2_S (1 h incubation) as detected by fluorescence microscopy using CN-BOT based tag-switch assay (C) or measured in gel from the cell lysates, using CN-Cy3 based tag-switch assay (D). Scale bar 20 μm. (E) Semi-quantification of in-gel fluorescence intensity. Experiments were performed in triplicates. Data are presented as mean ± S.D., *p < 0.01. (F–H) The effect of 2 μM auranofin or 100 μM Na_2_S treatments (1 h, 37 °C) on the intracellular persulfide levels in SH-SY5Y cells, measured by fluorescence microscopy using CN-BOT based tag-switch assay (F) or measured in gel from the cell lysates, using CN-Cy3 based tag-switch assay (G). Scale bar 20 μm. (H) Semi-quantification of in-gel fluorescence intensity. Experiments were performed in triplicates. Data are presented as mean ± S.D., *p < 0.01.

During optimization of the labelling protocol, we observed that persulfidation levels in cell lysates obtained using native conditions (HEN buffer pH 7.4, 1% protease inhibitor cocktail, 0 °C), decreased with incubation/extraction time (Fig. 6A and S10 in ESI†). We hypothesized that the decrease in persulfidation levels could be due to the activity of the Trx/TrxR/NADPH system during sample preparation. To test this hypothesis, we added 2 μM auranofin, an inhibitor of the Trx/TrxR/NADPH system,^40^–^42^ to the native lysis buffer, which led to preservation of the persulfidation levels (Fig. 6A). Furthermore, addition of Trx/TrxR/NADPH to the cell lysate led to immediate H_2_S release, as detected by the H_2_S electrode (Fig. 6B).

Next, we assessed how inhibition of the Trx/TrxR system affected intracellular protein persulfides levels. Cells (SH-SY5Y and BAE) were treated with auranofin (2 μM, 1 h) and then labelled with CN-BOT. Incubation with auranofin led to 1.8 ± 0.2 (BAE cells) and 2.1 ± 0.3 (SH-SY5Y cells) fold increases in fluorescence intensity (Fig. 6C and F) compared to control, which was enhanced further by combining auranofin with H_2_S. An overall increase in intracellular persulfidation was also observed in cell lysates (Fig. 6D, E, G and H) and the quantification of in-gel fluorescence signal matched nicely with the data observed by fluorescence microscopy.

**Fig. 7:**
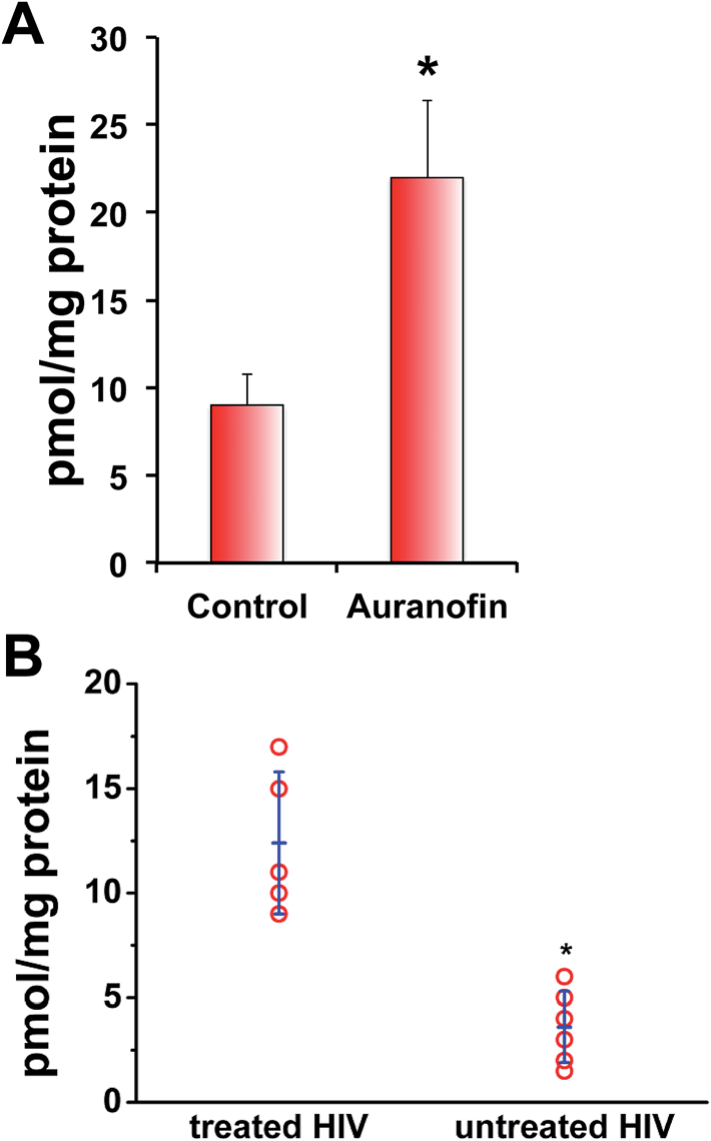
*In vitro* and *in vivo* sulfane sulphur levels are control by thioredoxin system. (A) Quantification of total sulfane sulfur levels in control and BAE cells treated with 2 μM auranofin for 1 h done by isotope dilution MS approach (see Fig. S11 in ESI†). Data are presented as mean values ± S.D. of *n* = 5, *p < 0.001. (B) Quantification of total sulfane sulfur levels in plasma samples from untreated and ART-treated HIV patients by isotope dilution MS approach. Data are presented as mean ± S.D. *p < 0.001 (see Fig. S12 and Table S1†).

Finally, we used an isotope dilution method mass spectrometry for quantification of the effect of auranofin inhibition of TrxR on intracellular persulfide levels. This method is based on the reaction of triphenylphosphines with sulfane sulfur (Fig. S10 in ESI†).^43^ BAEC lysates were prepared with probe **1** being added directly to the lysis buffer together with probe **2** (Fig. S10 in ESI†). Following protein precipitation, acetonitrile extracts were analysed by MS (Fig. S10 in ESI†). As observed using the tag-switch assay, sulfane sulfur levels increased in cells treated with auranofin with a total value of 9 ± 2 pmol mg^–1^ protein in control cells and 22 ± 5 pmol mg^–1^ protein in auranofin-treated cells (Fig. 7A).

The increased Trx levels is associated with certain disease states, such as rheumatoid arthritis, hepatitis C or HIV-1 infections.^44^–^46^ HIV-1 patients with high viral load have increased levels of circulatory Trx.^44^,^45^ We used plasma samples from HIV-1 patients with high viral load and compared their sulfane sulfur levels with those from HIV-1 patients treated with antiretroviral therapy (ART) that contain no detectable viral load. Significantly lower total sulfane sulfur levels were detected in patients with high viral load (and therefore high circulatory Trx levels)^44^,^45^ compared to ART-treated patients, confirming the role of Trx as a putative depersulfidase (Fig. 7B). Furthermore, there is a positive correlation between the viral load and the level of circulatory sulfane sulfur levels: the higher the viral load the lower the sulfane sulfur levels (Fig. S11 in ESI†). Although the actual role of lower persulfide levels in those HIV-1 patients awaits further investigation, this is the first *in vivo* evidence that Trx acts as depersulfidase.

## Conclusion

In conclusion, our work identifies Trx as a major regulator of intracellular persulfidation. Surprisingly, Trx showed almost an order of magnitude higher reactivity towards persulfides than towards disulfide analogs. The high reactivity of Trx towards LMW persulfides raises questions about the unusually high levels of LMW persulfides (>100 μM) recently reported.^32^ The identification of Trx as a depersulfidase provides a new perspective on persulfidation as an oxPTM of cysteine by revealing an enzymatic mechanism for reversing this posttranslational modification.

The improved tag-switch assay for facile persulfide labelling and subsequent in-gel detection or visualization by microscopy led to the observation that a significant portion of persulfidation is apparently localized in mitochondria. The use of the mitochondria targeted H_2_S donor, or the MST substrate d-cysteine, dramatically increased intracellular levels of persulfidation implying an important role for MST and mitochondria-derived H_2_S in protein persulfidation. Under oxidative stress conditions, formation of sulfenic acids is triggered which can react with H_2_S to form protein persulfides. These conditions also favour inhibition of the Trx system further prolonging the half-life of protein persulfides. The actual consequences of those steps remain to be elucidated. On one hand such a mechanism could be protective, as overoxidized persulfidated cysteine residues (P-SSO_3_^2–^)^23^,^25^ could be reduced by Trx system (similar like in the case of 3′-phosphoadenosine-5′-phosphosulfate reductase).^47^ Overoxidation of cysteine residues to form P-SO_3_^2–^, however, remains irreversible. On the other hand, excessive persulfidation could be inhibitory for some enzymes and prove detrimental in certain disease states. The improved tag-switch method for persulfide detection and herein reported observation that Trx regulates persulfide levels provide a good starting point for the future studies which will answer those questions and elucidate the actual mechanisms of H_2_S signalling.

## Materials and methods

### Materials

All chemicals were purchased from Sigma Aldrich. All buffers were prepared with nanopure water and treated with Chelex-100 resins to remove traces of metal ions. Na_2_S solutions were prepared and handled as recommended.^48^ NAP-SSH was prepared following the described synthetic protocol.^30^ (10-oxo-10-(4-(3-thioxo-3*H*-1,2-dithiol-5-yl)phenoxy)decyl)triphenylphosphonium bromide (AP39) was synthesized in-house as described.^37^

### Ultra-high resolution mass spectrometry

Mass spectrometry was performed on maXis 5G, Bruker Daltonik (Bremen, Germany), an ESI-ToF MS capable of resolution of at least 40.000 FWHM. Detection was in positive-ion mode. For the real-time measurements, NAP-SSH and Trx were mixed fast in the injection syringe (10 s) and then continuously injected into maXis (150 μL h^–1^). Spectra were recorded over 15 min time. Measurements were performed under native conditions, *i.e.* only in ammonium carbonate buffer pH 7.38. For the detection of Trx oxidation, Trx solutions were incubated with NAP-SSH or HSA-SSH for 2 min, mixed with acetonitrile (1 : 1, v/v) and formic acid (0.1% final concentration) and injected into MS.

### Preparation of cysteine persulfide

Recombinant human CSE (polymorphic variant S403) was expressed and purified as described previously.^49^ Twenty μg of CSE was added to 100 mM HEPES pH 7.4 containing 1 mM l-cystine. The mixture was incubated at 37 °C for 15 min and the enzyme was separated by ultrafiltration (centrifugation at 14 000 × *g* at 4 °C for 15 min) using a centrifugal filter with a molecular weight cutoff of 10 kDa (Carl Roth, Karlsruhe, Germany). Cysteine persulfide in the filtrate was quantified with the DTNB method, using an extinction coefficient *ε*_412_ of 14 150 M^–1^ cm^–1^. Concentrations of remaining unreacted cystine were calculated accordingly. Cysteine persulfide solutions were used immediately after filtration and prepared freshly before each experiment.

### Preparation of HSA-SSH

Persulfide of human serum albumin was prepared and its concentration quantified as reported previously.^27^

### Enzyme kinetics of human Trx with cysteine persulfide and HSA-SSH

Kinetics of the reaction of human Trx with cysteine persulfide was performed measuring NADPH oxidation on HP 8452A diode array spectrophotometer. As unreacted cystine could not be separated from cysteine persulfide, additional control measurements with respective cystine concentrations under otherwise identical conditions were performed. 1 μM human Trx, 10 nM TrxR and 250 μM NADPH were used in all measurements with various concentrations of the substrates. The absorption maximum of NADPH at 340 nm was monitored over a course of 5 min in intervals of 5 s. The initial rate of A_340_ decrease was fitted linearly using Origin® analysis software. Consumption of NADPH over time was determined using an NADPH extinction coefficient *ε*_340_ of 6020 M^–1^ cm^–1^. Rates of NADPH oxidation were plotted against the concentration of the mixture of cysteine persulfide and cystine or cystine alone, respectively. The data obtained were fitted using Michaelis–Menten equation in Origin® analysis software. An analogous set of experiments was performed using the persulfide derivative of HSA.

### Kinetics of the direct reaction of human Trx with cysteine persulfide and NAP-SSH

Kinetics of the reaction of thioredoxin with cysteine persufide was monitored on FP-8200 spectrofluorometer (Jasco, Germany) using an excitation wavelength of 280 nm and a maximal emission of 345 nm. In two separate sets of experiments either human or *E. coli* Trx (for comparison) were used. Concentration of the enzyme was kept at 1 μM while substrate concentrations ranged between 10 μM and 50 μM. Given pseudo first-order conditions, observed rate constants *k*_obs_ were obtained by fitting the decrease in emission at 345 nm at a given cysteine persulfide or cystine concentration using a first order exponential decay fit in Origin® analysis software. The *k*_obs_ were plotted against (CysSS^–^ + CysSSCys) or CysSSCys concentrations, and linearized. For comparison, an analogous set of measurements was performed using NAP-SSH and *E. coli* Trx.

### H_2_S detection

Kinetics of H_2_S release was followed amperometrically using a selective H_2_S electrode connected to Free Radical Analyzer (World Precision Instruments). Experiments were performed in 96-well plate in 200 μL volume. Electrode was calibrated using standard Na_2_S solutions.

### Spectral characteristics of free and protein-bound fluorescent dyes

To compare spectral characteristics of free CN-BOT and free CN-Cy3 with those of their HSA-bound counterparts, absorption and emission spectra of those species were recorded. Namely, the labelling was performed by blocking 200 μM HSA-SSH and 200 μM HSA-SH (as a control) with 10 mM MSBT for 1 h at 37 °C. After purification from excess blocking reagent by methanol/chloroform precipitation, samples were split in two aliquots and treated with 0.5 mM of CN-Cy3 and CN-BOT for 1 h at 37 °C, respectively. Excess free dye was removed by three steps of ultrafiltration (centrifugation at 14 000 × *g* for 15 min) using a centrifugal filter with a molecular weight cut-off of 3 kDa (Carl Roth, Karlsruhe, Germany). For this, the solution, which was concentrated about 10-fold after the individual centrifugation steps, was diluted to its original volume and the centrifugation was repeated. After the last centrifugation step, a methanol/chloroform precipitation was performed and the obtained protein pellets were re-dissolved in PBS. Absorption spectra were recorded at a HP 8452A diode array spectrophotometer, and fluorescence spectra were at a FP-8200 spectrophotometer (Jasco, Germany).

### Cell culture

SH-SY5Y cells (ECACC, Sigma Aldrich) were grown in Ham's F12 : DMEM (1 : 1) medium supplemented with 2 mM glutamine, 1% non-essential amino acids, and 10% fetal bovine serum at 37 °C and 5% CO_2_. BAE cells were grown in Ham's F12 medium supplemented with 2 mM glutamine, 1% non-essential amino acids, and 10% fetal bovine serum at 37 °C and 5% CO_2_.

### Mice

CSE^+/–^ and CSE^–/–^ mice were generated and characterized earlier (Ishii *et al.*, 2010). In this study, CSE^+/–^ males and females were bred to obtain CSE^+/+^, CSE^+/–^ and CSE^–/–^ littermates. The Berlin Animal Review Board approved all protocols that were conducted to American Physiological Society standards.

### CN-Cy3-based detection of persulfide levels in CSE^–/–^ and CSE^+/+^ mice

Organs (lungs, kidney, heart, liver, and brain) from CSE^–/–^ and CSE^+/+^ mice were isolated on ice, cleaned from connective tissue, and washed with ice-cold PBS. Immediately after isolation, the tissue was minced and homogenized on ice in HEN buffer (100 mM HEPES, 1 mM EDTA, 0.1 mM neocuproine, 1% SDS, pH7.4) supplemented with 20 mM MSBTA, 1% NP40 and 1% protease inhibitor cocktail, using Potter homogenizer. Homogenates were centrifuged at 10 000 × *g* for 20 min at 4 °C and clear supernatant was further incubated on ice for 1 h. 200 μL of supernatant samples were precipitated with water/methanol/chloroform mixture (v/v/v: 4/4/1) and centrifuged at 14 000 × *g* for 20 min at 4 °C. Obtained protein precipitates were dried under vacuum at 4 °C and stored at –80 °C. For improved tag-switch assay equal amounts of sample proteins (50 μg) were incubated with 100 μM Cy3-CN in 50 mM HEPES (pH 7.4) supplemented with 1.5% SDS, in dark, at 37 °C for 2 h, mixed with 4× non-reducing sample buffer (Bio-Rad, USA) and incubated for 30 min at 50 °C in dark. Samples were resolved on 10% SDS polyacrylamide gels in dark and immediately after short fixation and washing gels were scanned using ChemiDoc MP fluorescent imager (Bio-Rad, USA). Obtained images were semi-quantified using ImageJ software (NIH, USA).

### *In situ* fluorescence detection of intracellular persulfidation

Cells were grown in μ-dishes (35 mm, high) obtained from Ibidi® following manufacturer's instructions. The treatments with inhibitors, Na_2_S and AP39 were performed over 1 h. After treatments the cells were washed twice with warm sterile PBS. Fixation was carried out by incubation with ice-cold methanol at –30 °C for 20 min and subsequent permeabilization with ice-cold acetone at –30 °C for 5 min. The dishes were washed with PBS and incubated with 50 mM HEPES containing Triton (1%) and MSBT (10 mM) at RT overnight. After 3× washing the cells with PBS they were incubated with CN-BOT (25 μM) in PBS for 1 h at 37 °C. The cells were then washed 5× with PBS and stained with DAPI following manufacturer's recommendation. Images of 1024 × 1024 pixels were obtained using a LSM 780 confocal laser scanning system (Carl Zeiss MicroImaging) equipped with an Argon laser (458, 488, and 514 nm), a diode laser (405 nm), a DPSS-laser (561 nm) (LASOS Lasertechnik, Jena, Germany), mounted on an inverted Axio Observer Z1. The filter settings of the confocal scanner were: 514 nm excitation for Cy3 (MBS 458/561, filter 566–681 nm), 488 nm excitation for Alexa 488 (MBS 488/561/633, filter 493–543 nm) and 405 nm excitation for 40,6-diamidino-2-phenylindole hydro-chloride (MBS-405). A ×20 dry objective lens (numerical aperture 0.8) and a ×63 oil objective lens (numerical aperture 1.4) were used. Sequential scanning and appropriate pinhole settings minimized spectral bleed-through. For examination of co-localization of immunofluorescence, single optical sections at the same focus plane were taken separately and the three corresponding channels were merged into a 8-bit RGB tiff-file using confocal assistant software ZEN 2010.

### Mitochondrial visualization and co-localization studies

For co-localization studies, cells were transfected with CellLight® Mitochondria-RFP, BacMam 2.0 obtained from ThermoFisher Scientific® following the manufacturer's instructions. It is a fusion construct of the leader sequence of E1 alpha pyruvate dehydrogenase and TagRFP, providing specific targeting of cellular mitochondria-RFP through transient transfection with the insect virus baculovirus. After transfection and following the expression of mitochondria-RFP overnight, the cells were treated with AP39, D-Cys and auranofin for 1 h at 37 °C. Cells were fixed with 4% formaldehyde for 45 min, washed with PBS and incubated with 50 mM HEPES containing Triton (1%) and MSBT (10 mM) at room temperature overnight. After washing with PBS (3×), the fixed cells were incubated with CN-BOT (25 μM) in PBS for 1 h at 37 °C. The cells were then washed 5× with PBS and stained with DAPI following manufacturer's recommendation.

### Detection of protein persulfidation in cell lysates

Cells were grown in T-75 cell culture flasks. After treatments, the cells were washed twice with warm sterile PBS. Lysis was performed by addition of 800 μL HEN buffer (50 mM HEPES, 0.1 mM EDTA, pH 7.4) containing SDS (1.5%), NP-40 (1%), protease inhibitor cocktail (1%) and MSBT (10 mM) to a T-75 flask. Cells were incubated for 10 min on ice with occasional scraping of the flask surface with a cell scraper. The lysates were transferred to microcentrifugation tubes and incubated at 37 °C for 1 h. Protein precipitation was performed using H_2_O/CHCl_3_/MeOH (4/1/4) precipitation, with subsequent vigorous mixing and centrifugation (20 000 × *g*, 20 min, 4 °C). The supernatant was discarded and the precipitate was dried. The pellet was resuspended in 300 μL of 50 mM HEPES containing 3% SDS. Lysates were stained with CN-Cy3 (60 μM) for 1 h at 37 °C. SDS-PAGE was run under non-reducing conditions and the gels were recorded on a ChemiDocTM MP System with the Cy3 setup.

### Isotope dilution mass spectrometry for the quantification of sulfane sulfur

The reagents were synthesized as described previously.^40^ 1 mM probe **1** and 30 μM probe **2** (final concentrations) were added into the HEN lysis buffer which also contained 1.5% SDS and 1% protease inhibitors. 200 μL of this mixture was added directly into the flask and cells scraped from the flask. Cell lysates were then incubated 1 h at 37 °C with occasional vigorous vortexing. Protein concentrations were determined in the samples and proteins were then precipitated by the addition of acidified acetonitrile. Samples were vortexed and centrifuged, and the supernatants were taken for the MS analysis.

### HIV patients

11 HIV-1-infected patients were included in the study. The study was approved by the Ethics Committee of the Medical Faculty of the Friedrich-Alexander-Universität Erlangen-Nürnberg and the patients gave informed consent. The patient characteristics are shown in the Table S1.† Five patients were on antiretroviral treatment with a suppressed viral load of <20 copies per ml and a median CD4 count of 364/μl (range 309–955). Six patients were untreated with a median HIV-1-viremia of 10 700 copies per ml (range 2600–5 400 000) and a median CD4 count of 436 (range 143–684). Sulfane sulfur levels were expressed as pmol mg^–1^ of plasma protein.

## Conflict of interest

MW, MEW and the University of Exeter have patent applications relating to the therapeutic and agricultural use of H_2_S donors including AP39.

## Author contribution

RW, CO, JM and MRF performed most of the experiments. SW synthesized and characterized CN-BOT. AM synthesized and characterized CN-Cy3. IA, MG and II provided CSE^–/–^ mice and helped with tag-switch method evaluation in animal tissue. EG synthesized NAP-SSH. MX provided isotope-dilution MS reagents and helped with data planning and analysis. EGH and TH collected plasma samples from HIV patients and performed data analysis. RT, MW and MEW synthesized AP39 and helped in data analysis. PKY and RB purified recombinant human CSE and helped with data analysis and manuscript writing. All authors contributed in manuscript writing. MRF designed the study, analysed the data and wrote the manuscript.

## Supplementary Material

Supplementary information
