# Supplementary material for: Improved tag-switch method reveals that thioredoxin acts as depersulfidase and controls the intracellular levels of protein persulfidation
Source: Chem Sci. 2016 Feb 9;7(5):3414–26. doi: 10.1039/c5sc04818d (PMC4845716; doi:10.1039/c5sc04818d)

Electronic Supplementary Material (ESI) for Chemical Science.
This journal is © The Royal Society of Chemistry 2016

Electronic Supporting Information

Improved Tag-Switch Method Reveals that Thioredoxin Acts as Depersulfidase and Controls the Intracellular Levels of Protein Persulfidation

Rudolf Wedmann,[1#] Constantin Onderka,[1#] Shengwei Wei,[1] István András Szijártó,[2] Jan Lj. Miljkovic,[1] Aleksandra Mitrovic,[3] Mike Lange,[1] Sergey Savitsky,[1] Pramod Kumar Yadav,[4] Roberta Torregrossa,[5,6] Ellen G. Harrer,[7] Thomas Harrer,[7] Isao Ishii,[8] Maik Gollasch,[2] Mark E. Wood,[6] Erwan Galardon,[9] Ming Xian,[10] Matthew Whiteman,[5] Ruma Banerjee,[4] Milos R. Filipovic[1,11*]

[1]Department of Chemistry and Pharmacy, Friedrich-Alexander University of Erlangen-Nuremberg, Erlangen, Germany;

[2]Charité Campus Virchow, Nephrology/Intensive Care, Berlin, Germany;

[3]Department of Chemistry, University of Belgrade, Belgrade, Serbia;

[4]Department of Biological Chemistry, University of Michigan, Ann Arbor, USA;

[5]University of Exeter Medical School, St. Luke’s Campus, Exeter, UK;

[6]Biosciences, College of Life and Environmental Sciences of Biosciences, University of Exeter, Streatham Campus, Exeter, Devon, UK;

[7]Infectious Diseases Section, Department of Internal Medicine 3, Universitätsklinikum Erlangen, Friedrich-Alexander-University Erlangen-Nürnberg;

[8]Department of Biochemistry, Graduate School of Pharmaceutical Sciences, Keio University, Tokyo, Japan;

[9]UMR CNRS 8601, Université Paris Descartes, Sorbonne Paris Cité, Paris, France;

[10]Department of Chemistry, Washington State University, Pullman, USA;

[11]IBGC, UMR CNRS 5095, Université de Bordeaux, Bordeaux, France.

Table of Contents:

**1-Supplemental synthetic schemes**	**3**
**2-Summary of Generic Analytical and Chromatographic Conditions**	**4**
**3-Synthetic procedures**	**5**
**4-Additional References**	**8**
**5-Supporting Figures**	**9**
**6-Supporting Tables**	**21**
**7-Product characterization**	**22**

1-Supplemental synthetic schemes

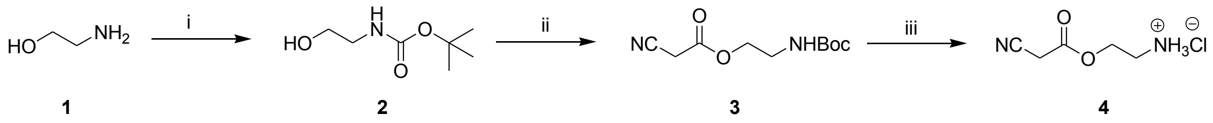

Scheme S1: *Reagents and conditions*: (i) ethanolamine, Boc_2O, TEA, DCM, 0°C, 2 h; (ii) cyanoacetic acid, DCC, THF, 0°C to RT, 24 h; (iii) HCl gas passed through the ether solution of cmpd **3,** 65 % overall yield.

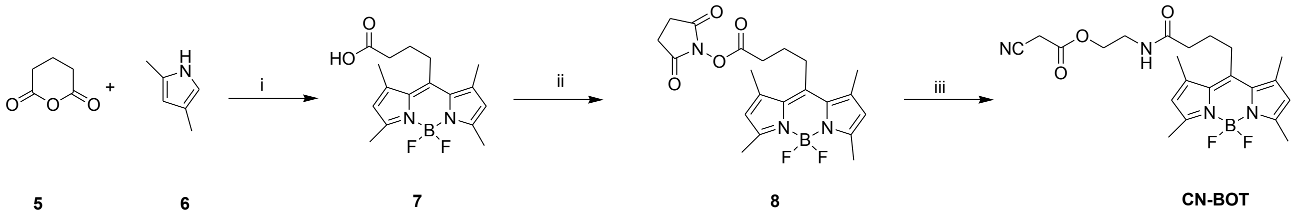

Scheme S2: *Reagents and conditions*: (i) $BF_3{\cdot}OEt_2$, THF, reflux for 5 h, RT for 24 h, 16 %; (ii) NHS, DCC, THF, RT, 5 h, 99 %; (iii) **4**, TEA, DMF, RT, 24 h, 85 %.

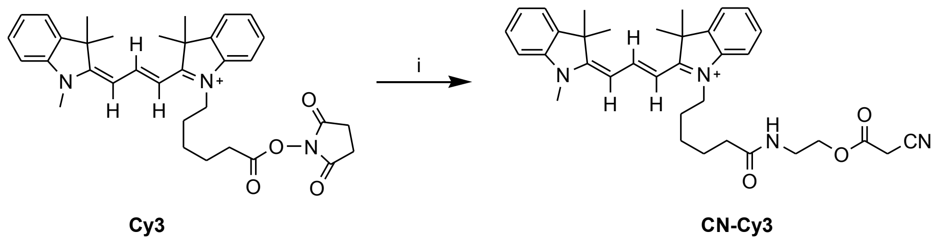

Scheme S3: *Reagents and conditions*: (i) **4**, TEA, DMF, RT, 24 h, 80%.

2-Summary of Generic Analytical and Chromatographic Conditions

All reactions were performed in flame-dried glassware under a nitrogen atmosphere with dry solvents, unless noted otherwise. All reagents and starting materials were purchased from: Sigma Aldrich, Alpha Aesar or Acros Organic and used without further purification.

Flash column chromatography was carried out using Merck 60 Kieselgel (230–400 mesh) under pressure. Analytical thin layer chromatography (TLC) was performed on aluminum plates pre-coated with Merck silica gel 60 F_{254}, and visualized by ultra-violet irradiation or by staining with aqueous acidic ammonium hexamolybdate, or aqueous acidic potassium permanganate solutions as appropriate.

^{1}H, ^{13}C nuclear magnetic resonance (NMR) spectra were obtained as either $CDCl_3$, CD_3OD or DMSO-*d*6 solutions and recorded at 300 MHz, 75 MHz on Bruker Avance DPX 300 NMR spectrometer. Chemical shifts are quoted in parts per million (δ) referenced to the appropriate deuterated solvent employed. Multiplicities are indicated by s (singlet), d (doublet), t (triplet), q (quartet), quin (quintet), m (multiplet), br (broad) or combinations thereof. Coupling constant values are given in Hz. High resolution mass spectrometry was carried out on a ESI- UHR-*TOF* (Bruker Daltonics, Bremen (Germany)).

Data were compared with literature data for compounds that had been previously reported.

3-Synthetic procedures

Preparation of 2-aminoethyl 2-cyanoacetate hydrochloride, (4)[1]

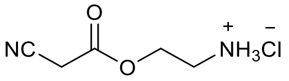

Ethanolamine (**1**) (4.90 g, 0.08 mol), TEA (6.28 g, 0.06 mol) and DCM (123 mL) were added to a flame-dried flask, under inert atmosphere. The mixture was cooled in an ice-water bath and a solution of di*tert*butyl dicarbonate (13.6 g, 0.06 mol) in DCM (5 ml) was added within 5 minutes. After the additional stirring for 2 hours, the reaction mixture was washed with 1M HCl at 0°C, 10% $NaHCO_3$ and organic layer dried over anh. $MgSO_4$**.** After the removal of the solvent product (**2**) was obtained as an oily liquid. Compound (**2**) was further dissolved in dry THF (130 mL) and cyanoacetic acid (42.0 g, 0.5 mol) was added at 0°C. After complete dissolution, *N,N'*-Dicyclohexylcarbodiimide (DCC) (12.7 g, 0.06 mol) in dry THF (10 mL) was added over 2 minute period of time and the reaction mixture was stirred overnight. Obtained precipitate was filtered off and thoroughly washed with ether. Through ether filtrate containing the desired product (**3**) HCl gas was passed in order to remove the protecting group. The precipitate was filtered off giving 2-aminoethyl 2-cyanoacetate hydrochloride (**4**) (6.6 g, 65%, based on Boc_2O) as a yellow solid.

^{1}H NMR (DMSO, 300 MHz): 8.26 (bs, 3H, NH_3^+), 4.34 (t, J = 5.1Hz, 2H), 4.09 (s, 2H), 3.06 (m, 2H); ^{13}C NMR ($CDCl_3$, 75 MHz) δ 164.4, 114.9, 62.0, 37.5, 24.9; HRMS (ESI-MS): calculated for $C_5H_8N_2O_2$ 129.0658, found 129.0657.

Preparation of 4-(4,4-Difluoro-1,3,5,7-tetramethyl-4-bora-3a,4a-diaza-s-indacene-8-yl)-butyric acid, (7)

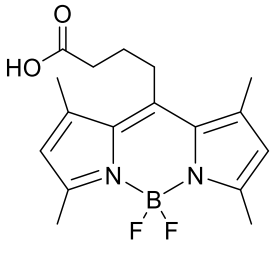

Glutaric acid anhydride (**5**) (0.31 g, 2.68 mmol), dry THF (45 mL), 2,4-dimethylpyrrole (**6**) (0.420 g, 4.41mmol), and $BF_3·OEt_2$ (0.77 g, 5.40 mmol) were sequentially added to a nitrogen-flushed

two-necked flask. The mixture was heated to reflux for 5 h under nitrogen. After the mixture was cooled to room temperature, TEA (1.82 g, 17.9 mmol) and $BF_3{\cdot}OEt_2$ (1.92 g, 13.5 mmol) were added slowly. The reaction mixture was stirred under nitrogen at room temperature for 24h. The resulting slurry was then extracted with DCM (3 x 150 mL) and combined organic layer washed with water (2 x 20 mL), dried over anhydrous Na_2SO_4 and evaporated. The obtained dark oil was purified by chromatography on silica gel (elution with hexane/ethyl acetate 2:1) to give 143 mg (16%) of BODIPY-butyric acid (**7**) as a dark red solid.

^{1}H NMR ($CDCl_3$, 300 MHz) δ 6.06 (s, 2H, pyrrole-H), 3.02 (t, 2H, $-CH_2$, *J* = 8.6 Hz), 2.52 (t, 2H, -CH_2, *J* = 8.6 Hz), 2.43 (s, 6H, $-CH_3$), 2.34 (s, 6H, $-CH_3$), 1.92-1.98 (m, 2H, $-CH_2$); ^{13}C NMR ($CDCl_3$, 75 MHz) δ 177.6, 154.3, 144.6, 140.4, 131.4, 121.9, 33.8, 27.4, 16.3, 14.4; HRMS (ESI-MS): $[M-H]^-$ calculated for $C_{17}H_{20}BF_2N_2O_2$ 333.1575, found 333.1580.

Preparation of 4-(4,4-Difluoro-1,3,5,7-tetramethyl-4-bora-3a,4a-diaza-s-indacene-8-yl) butyric acid NHS ester, (8)[2]

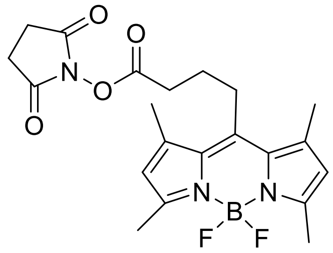

Compund (**7**) (0.07 g, 0.21 mmol) and N-hydroxysuccinimide (0.025 g, 0.23 mmol) were dissolved in anhydrous THF (3 mL). A solution of dicyclohexylcarbodiimide (0.025 g, 0.25 mmol) in anhydrous THF (1 mL) under dried condition was added to the previous solution slowly. Reaction mixture was agitated at room temperature for 5 h after what the obtained white precipitate was removed as a byproduct. The filtrate was evaporated under vacuum and the residue was purified by silica gel column chromatography with dichloromethane as eluent to give the desired red product (**8**) (0.09 g, yield 99 %).

^{1}H NMR ($CDCl_3$, 300 MHz): 6.05 (s, 2H), 3.10–3.04 (m, 2H), 2.83 (s, 4H), 2.79–2.77 (m, 2H), 2.50 (s, 6H), 2.40 (s, 6H), 2.09–2.03 (m, 2H); ^{13}C NMR ($CDCl_3$, 75 MHz) δ 169.0, 167.8, 154.4, 144.0, 140.4, 131.3, 122.6, 121.9, 30.9, 27.0, 26.1, 25.5, 20.5, 16.4, 14.4; HRMS (ESI): calculated for $C_{21}H_{24}BF_2N_3O_4Na$ ($M+Na^+$): 454.1726; $C_{21}H_{24}BF_2N_3O_4K$ 470.1465, found: 454.1714 and 470.1463.

Preparation of CN-BOT[3]

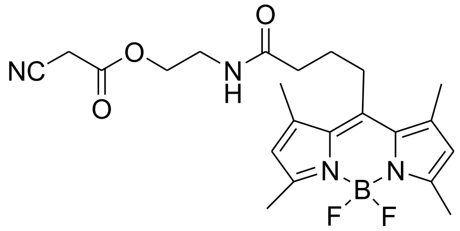

2-aminoethyl 2-cyanoacetate hydrochloride (**4**) (39.05 mg, 0.24 mmol) and TEA (49.9 μL, 0.36 mmol) were mixed in dry DMF (2.5 mL), to this solution compound (**8**) (52 mg, 0.12 mmol) was added and the mixture stirred for 24 h at room temperature under an argon atmosphere. The reaction mixture was quenched with saturated NH_4Cl solution and then poured into ice-water to form the precipitate. The crude compound was purified by column chromatography on Al_2O_3 (neutral) eluting by *gradient* elution with cyclohexane – dichloromethane system (1:2 to 0:1) to give **CN**-**BOT** (*46 mg, 85%)

^{1}H-NMR (300 MHz, $CDCl_3$) δ 6.05 (s, 2H), 3.73 (t, J = 4.8 Hz, 2H), 3.42 (s, 2H), 3.02 (q, J= 3.0 Hz, 2H), 2.51(s, 6H), 2.42 (s, 6H), 2.3 (t, J = 4.8Hz, 2H), 1.98 (t, J = 8.1Hz, 2H), 1.83 (m, 2H); HRMS (ESI): calculated for $C_{19}H_{26}BF_2N_3O_2Na$ $(M\text{-}COCH_2CN\text{+}H\text{+}Na)^+$: 400.1984; found: 400.1971.

**Solvent is present as minor impurity (NMR spectra).*

Preparation of CN-Cy3

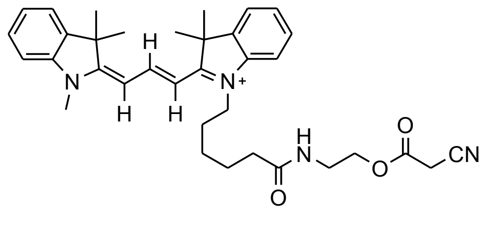

To a stirred solution of **Cy3** (5 mg, 0.008 mmol) in anhydrous DMF (200 μL) freshly recrystallized 2-aminoethyl 2-cyanoacetate (**4**) (1.7 mg, 0.013 mmol) was added. Then trimethylamine (0.02 mmol, 2.3 μL) was added and the reaction mixture stirred overnight at room temperature. The solvent was removed under vacuum and the residue purified by flash chromatography eluting with 100 % EtOAc and 2% to 10 % MeOH/$CHCl_3$ to afford product **CN-Cy3** as a purple film (*3.8 mg, 80 %).

^{1}H ($CDCl_3$, 500 MHz): δ 8.93 (t, 1H, *J* = 5 Hz); 8.42 (t, 1H, *J* = 10 Hz); 7.43-7.35 (m, 4H); 7.28-7.21 (m, 4H); 7.14 (dd, 1H, *J* = 15, *J* = 10 Hz), 7.02 (d, 1H, *J* = 15 Hz), 4.33 (t, 2H, *J* = 5 Hz), 4,13 (t, 2H, *J* = 7.5 Hz), 4.02 (s, 2H), 3.80 (s, 3H), 3.57-3.53 (m, 2H), 2.46 (t, 2H, *J* = 7.5 Hz), 1.93-1.83 (m, 4H), 1.73 (s, 6H), 1.72 (s, 6H), 1.70-1.58 (m, 2H).

**Diethyl ether is present as minor impurity (NMR spectra).*

4-Additional References

(1) Mcardle, C. B., et al. Activated methylene reagents and curable compositions prepared therefrom, **2009**, WO2009053482 A3.

(2) a) Zhang Z.X., Gao P.F, Guo X.F., Wang H., Zhang H.S. *Anal Bioanal Chem* **2011,** *401*, 905–1914; b) Meng Q., Yu M., Zhang H., Ren J., Huang D. *Dyes and Pigments* **2007**, *73*, 254-260.

(3) Li Z., Mintzer E., Bittman R. *J Org Chem* **2006**, *71*, 1718–1721.

5-Supporting Figures

Figure S1. Analysis of the MS data shown in the Figure 1A-C.

Figure S2. Analysis of the MS data shown in Figure 1D-F.

Figure S3. H_2S release form NAP-SSH treated with Trx.

Figure S4. MS analysis of the reaction mixture containing 20 μM Trx and 40 μM HSA-SSH reveals a complete oxidation of Trx.

Figure S5. Plot of k_{obs} vs. concentration of the used LMW species for the reaction with Trx (*E. coli*).

Figure S6. UV-vis and fluorescence spectral properties of HSA-SSH labelled with improved tag-switch method probes, CN-Cy3 and CN-BOT.

Figure S7. Comparison of tag-switch labelling (CN-Biotin) with improved tag-switch labelling (CN-Cy3).

Figure S8. Validation of CN-BOT based tag-switch method.

Figure S9. Inhibition of H_2S production leads to decrease of persulfidation in the cell lysates, as observed by improved CN-Cy3 based tag-switch assay.

Figure S10. Isotope dilution MS method for sulfane sulphur quantification.

Figure S11. Sulfane sulphur levels vs. viral load.

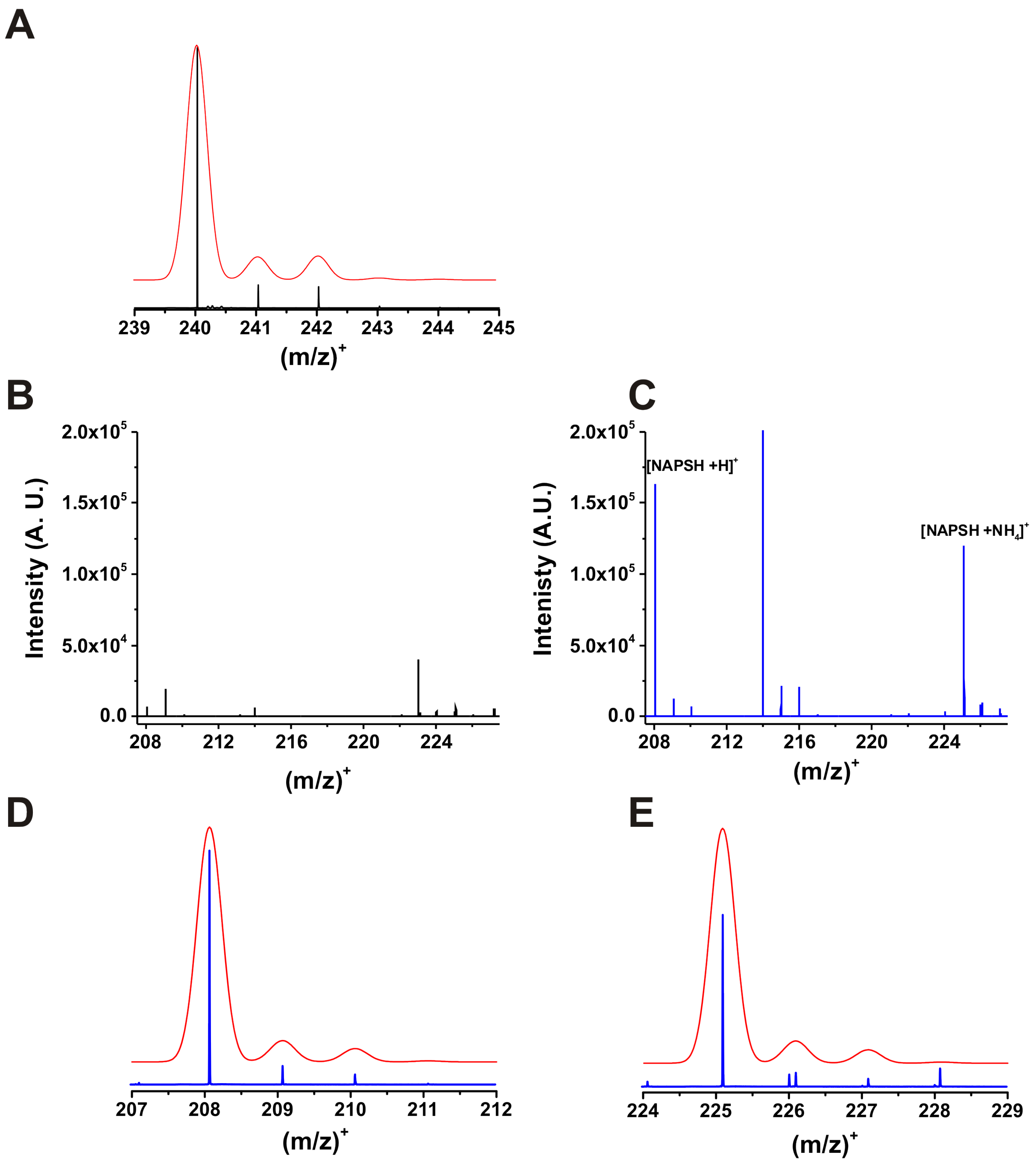

Figure S1. Analysis of the MS data shown in the Figure 1A-C. A) Observed (black) and simulated (red) isotopic distribution for NAP-SSH (*m/z* 240.0347; calculated 240.0359). B-C) MS spectra at t=0 (B) and t=5 min (C) of the reaction mixture containing 50 μM NAP-SSH and 50 μM Trx (*E. coli*) in ammonium carbonate buffer pH 7.38, indicate formation of NAPSH which ionizes as $[NAPSH + H]^+$ and $[NAPSH + NH_4]^+$. D) Observed (black) and simulated (red) isotopic distribution for $[NAPSH + H]^+$ (*m/z* 208.0623; calculated 208.0638). E) Observed (blue) and simulated (red) isotopic distribution for $[NAPSH + NH_4]^+$ (225.0883; calculated 225.0904).

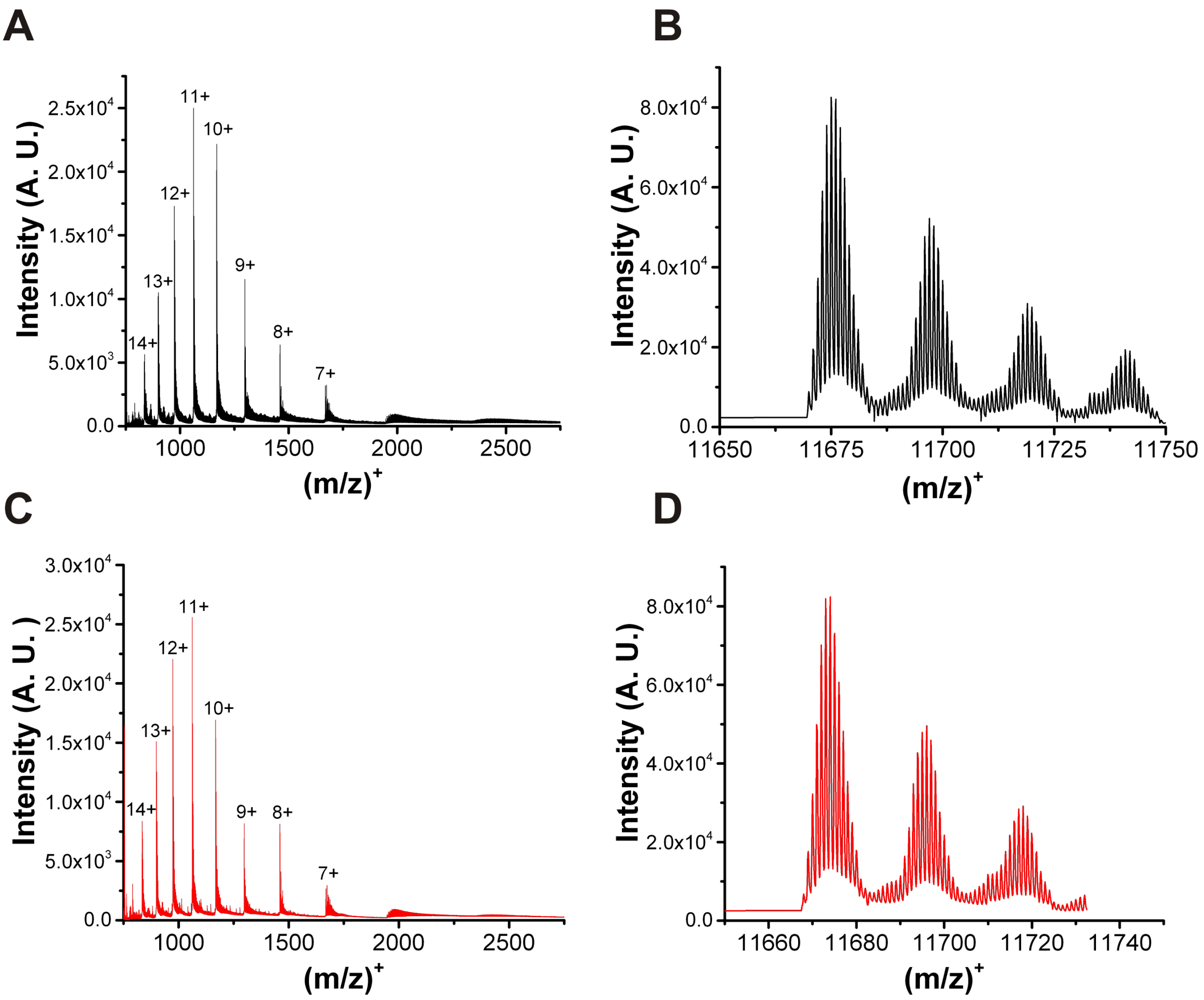

Figure S2. Analysis of the MS data shown in Figure 1D-F. A) Mass spectrum of fully reduced Trx. B) Deconvoluted MS spectrum shows the parent peak which corresponds to $[Trx_{red} + H]^+$ and subsequent peaks which correspond to the addition of 1 to 3 Na^+ ions. C) Mass spectrum of the Trx after 2 min of incubation with NAPSSH. D) Deconvoluted spectra which corresponds to $[Trx_{ox} + H]^+$ followed by the peaks which correspond to the addition of 1 to 3 Na^+ ions.

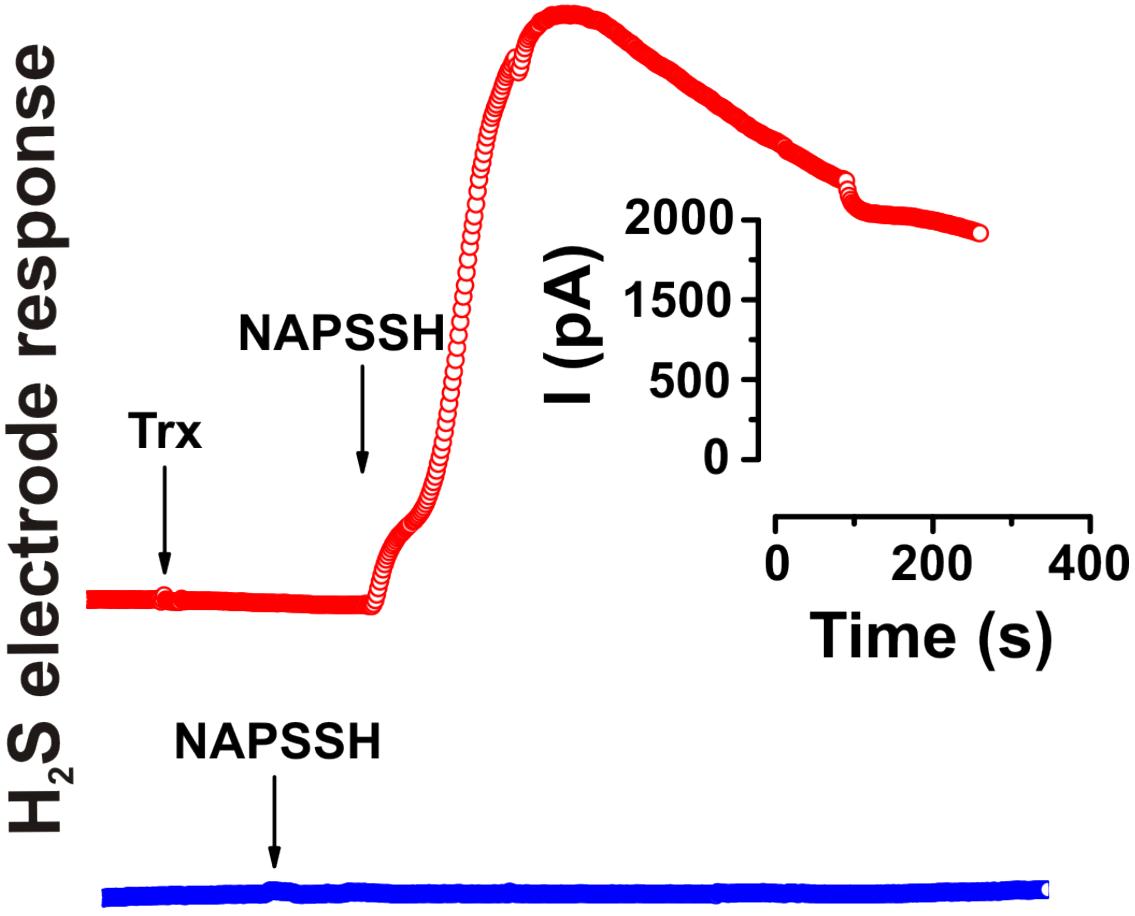

Figure S3. H_2S release form NAP-SSH treated with Trx. 100 μM NAP-SSH was added into PBS solution (pH 7.4) with H_2S electrode immersed in it (blue circles). No significant change in the electrode response could be observed. When 10 μM Trx was added into PBS solution (pH 7.4) with H_2S electrode immersed in it (red circles) no significant change in the electrode response could be observed either. However, subsequent injection of 100 μM NAP-SSH led to an immediate electrode response which correlates with the amount of released H_2S.

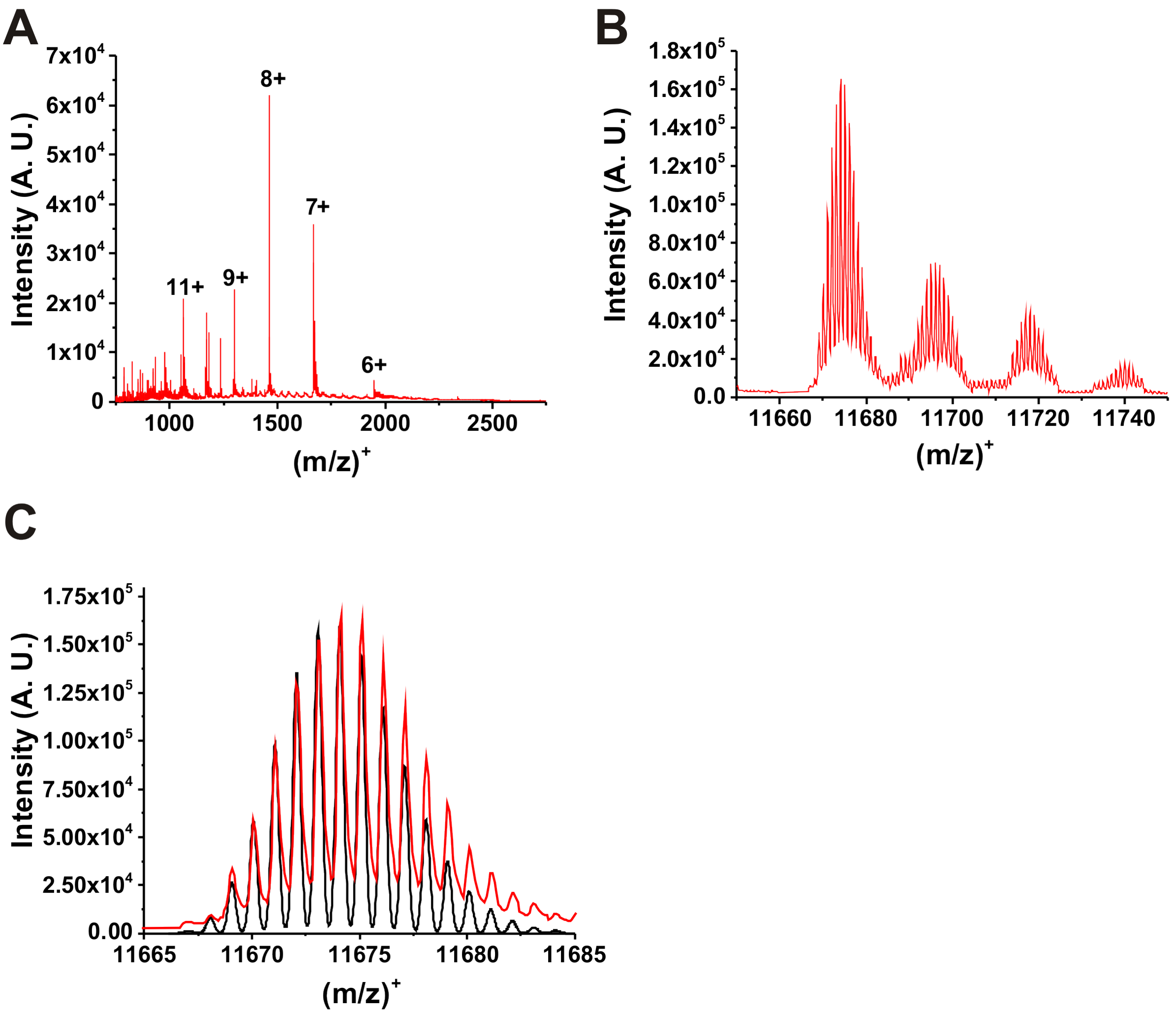

Figure S4. MS analysis of the reaction mixture containing 20 μM Trx and 40 μM HSA-SSH reveals a complete oxidation of Trx. A) Full MS spectrum showing differently charged Trx species. Due to the high ionizability of Trx and lower charge/molecule ratio, it was not possible to deconvolute the spectra of HSA in the same reaction mixture. HSA can be noticed in the spectrum as a regular pattern of signals in *m/z* 1500-2000 range. B) Deconvoluted spectrum showing $[Trx_{ox} + H]^+$, $[Trx_{ox} + Na]^+$, $[Trx_{ox} - H + 2Na]^+$ and $[Trx_{ox} - 2H + 3Na]^+$. C) Observed isotopic distribution (red) and simulated isotopic distribution for $[Trx_{ox} + H]^+$.

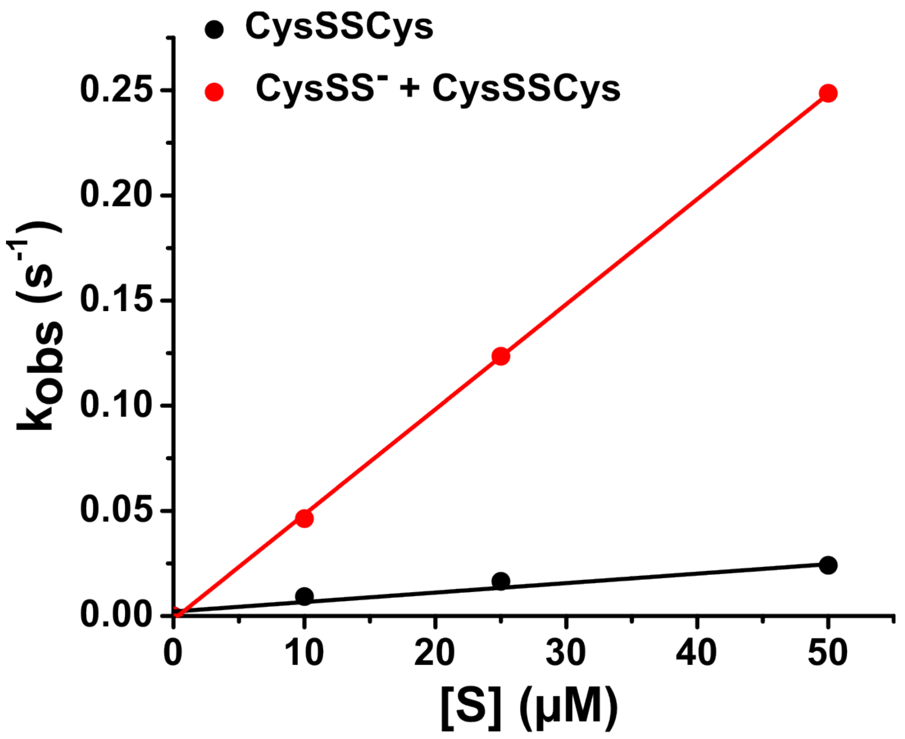

Figure S5. Plot of k_{obs} vs. concentration of the used LMW species for the reaction with Trx (*E. coli*). Reaction was followed fluorimetrically measuring conformational changes induced in Trx due to the cysteine oxidation (See Figure 2F).

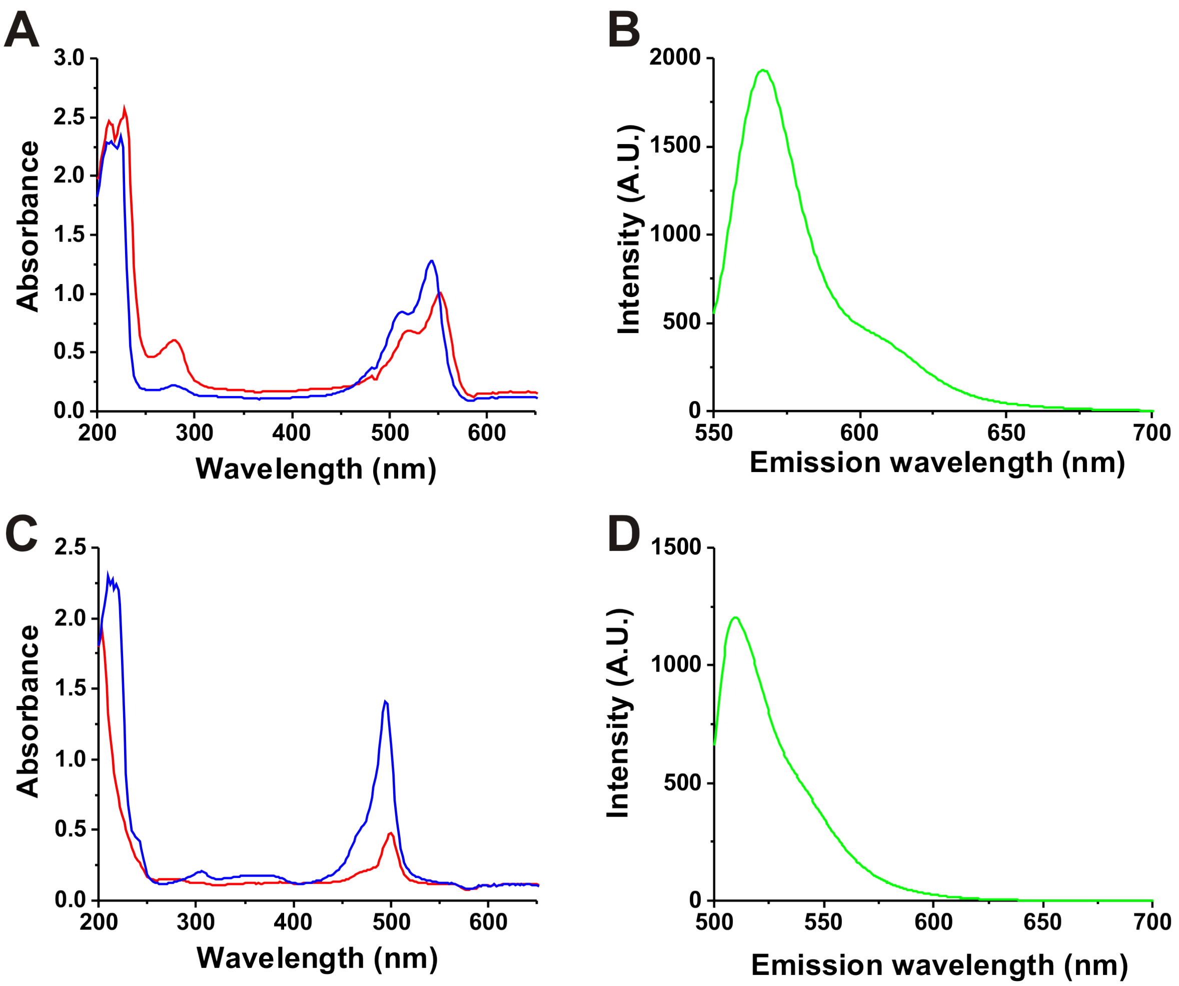

Figure S6. UV-vis and fluorescence spectral properties of HSA-SSH labelled with improved tag-switch method probes, CN-Cy3 and CN-BOT. A) UV-vis spectra of 67 μM HSA-SSH labelled with CN-Cy3 (red) and 75 μM free CN-Cy3 (blue). B) Emission spectrum of 100 μM HSA-SSH labelled with CN-Cy3 (λ_{ex} 542 nm). C) UV-vis spectra of 67 μM HSA-SSH labelled with CN-BOT (red) and 51 μM free CN-BOT (blue). D) Emission spectrum of 100 μM HSA-SSH labelled with CN-BOT (λ_{ex} 494 nm).

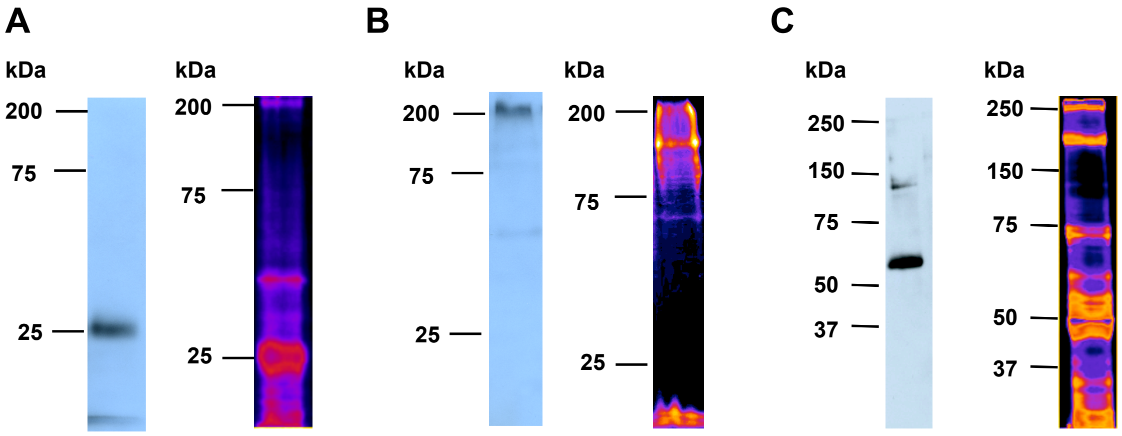

Figure S7. Comparison of tag-switch labelling (CN-Biotin) with improved tag-switch labelling (CN-Cy3). A) Human red blood cell lysates were labelled with either CN-Biotin and analysed by Western Blot or labelled with CN-Cy3 and in-gel fluorescence was detected. The same protein load is used for both experiments (20 μg). B) Proteins of red blood cell membranes visualized either by Western Blot or in-gel fluorescence. C) *D. melanogaster* head homogenates were labelled for persulfidation using either CN-Biotin or CN-Cy3 in the last step. The same protein amount was loaded (10 μg) in two separate gels and the bands visualized accordingly.

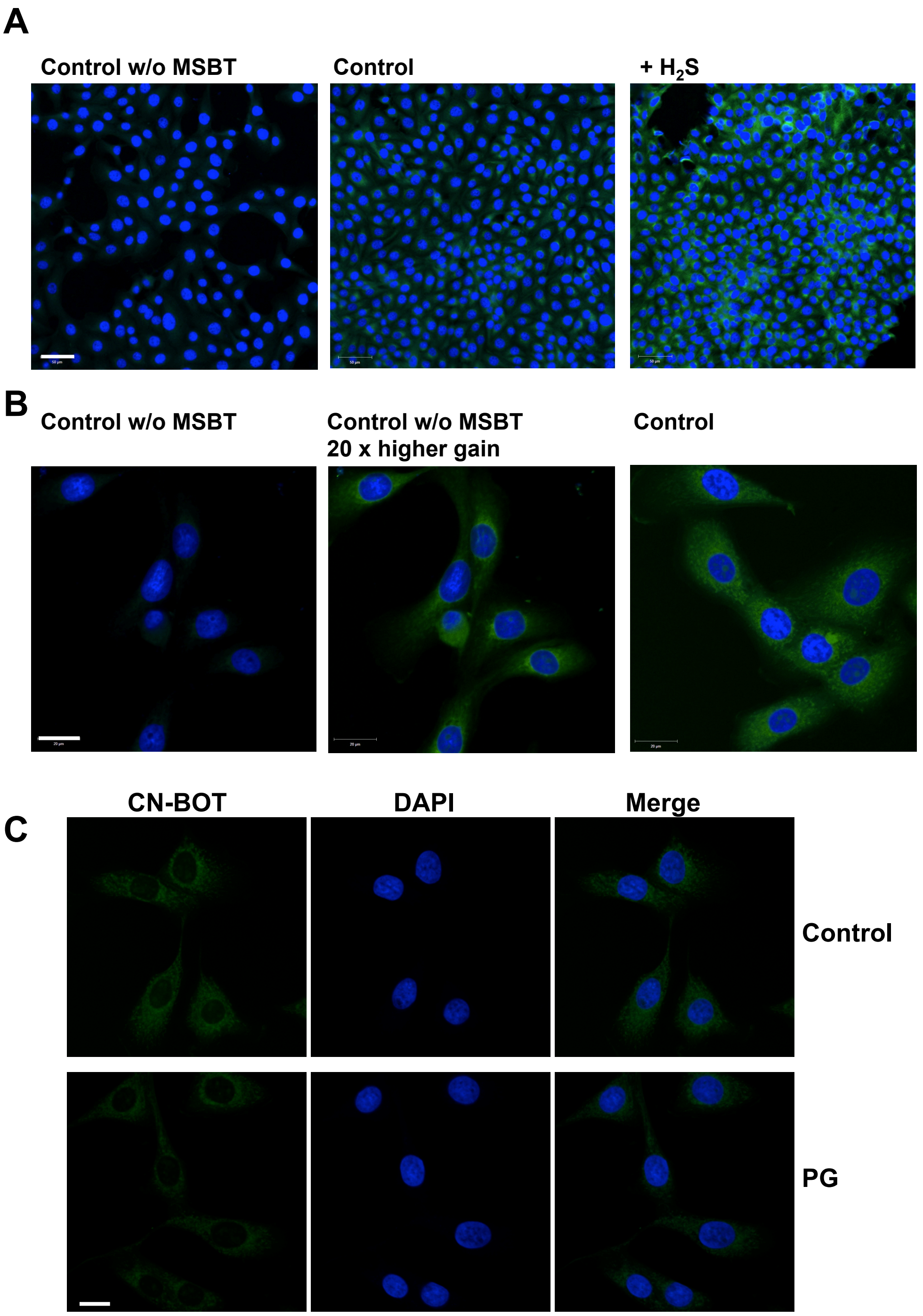

Figure S8. Validation of CN-BOT based tag-switch method. A) BAECs were fixed with methanol, delipidated with acetone and incubated with or without MSBT prior the exposure to CN-BOT. No detectable signal could be seen in the cells not treated with MSBT under the recording conditions where signal could be observed in the control cells and an increase of the signal in the cells treated with H_2S. Scale bar 50 μm. **B)** When laser intensity was increased 20 fold the nonspecific signal could be seen even in the cells treated only with CN-BOT. However, the intensity is still smaller than the one observed in the control cells recorded under normal condition. Even more, the pattern of the fluorescence is also different indicating that there is a weak nonspecific binding of the dye to the cell preparations. Scale bar 20 μm. **C)** BAECs incubated with 2 mM propargylglycine (PG) for 1 h show significantly lower intracellular persulfide levels (lower lane). Scale bar 10 μm.

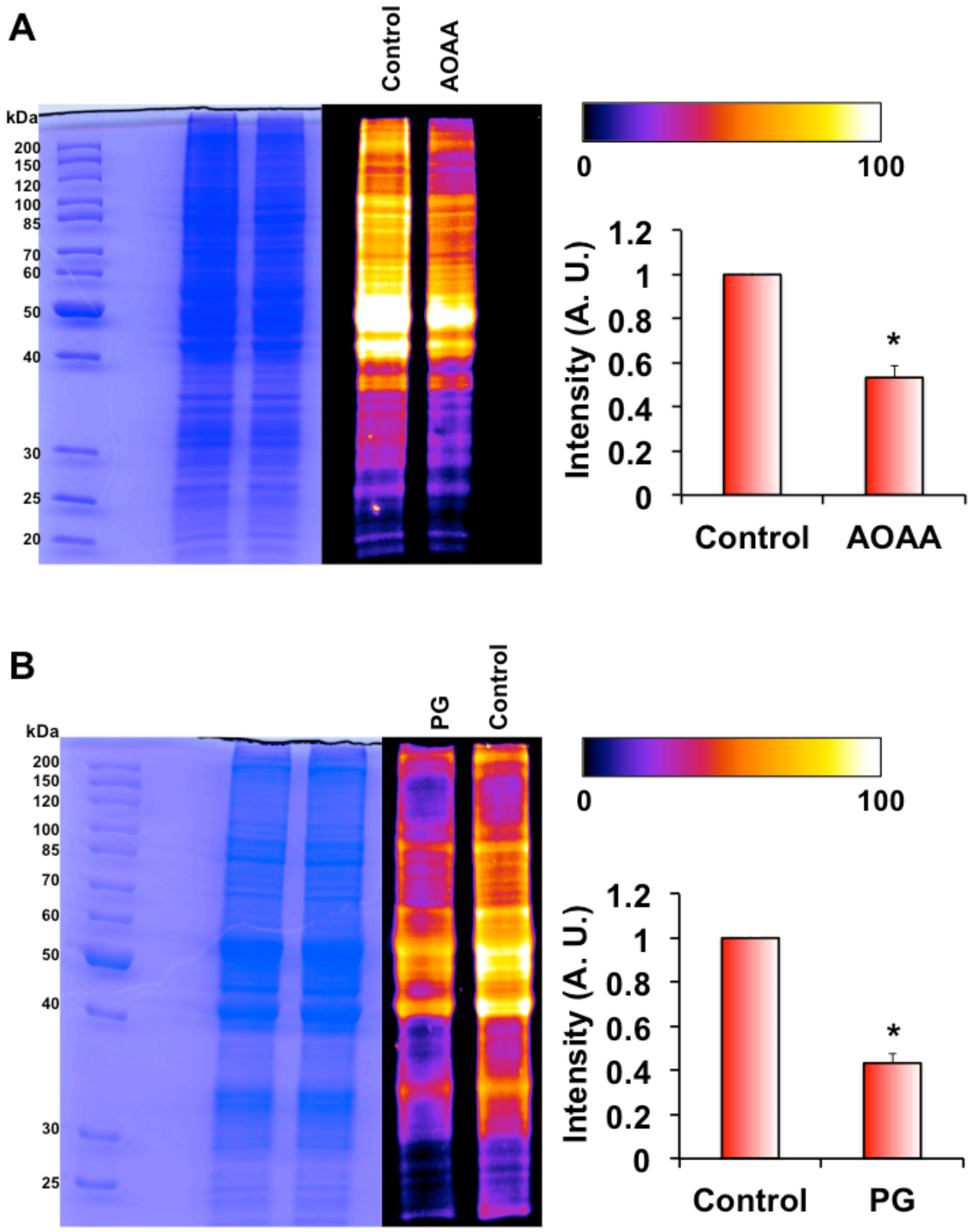

Figure S9. Inhibition of H_2S production leads to decrease of persulfidation in the cell lysates, as observed by improved CN-Cy3 based tag-switch assay. A) SH-SY5Y cells were incubated with 2 mM AOAA for 4 h, mean ± S.D., n=3, *p<0.001. B) BAEC were incubated with 2 mM PG for 4 h, mean ± S.D., n=3, *p<0.001. Pseudo-colouring of the gels was used to better visually indicate the differences in the signal intensities. Colour scale is given in the figure.

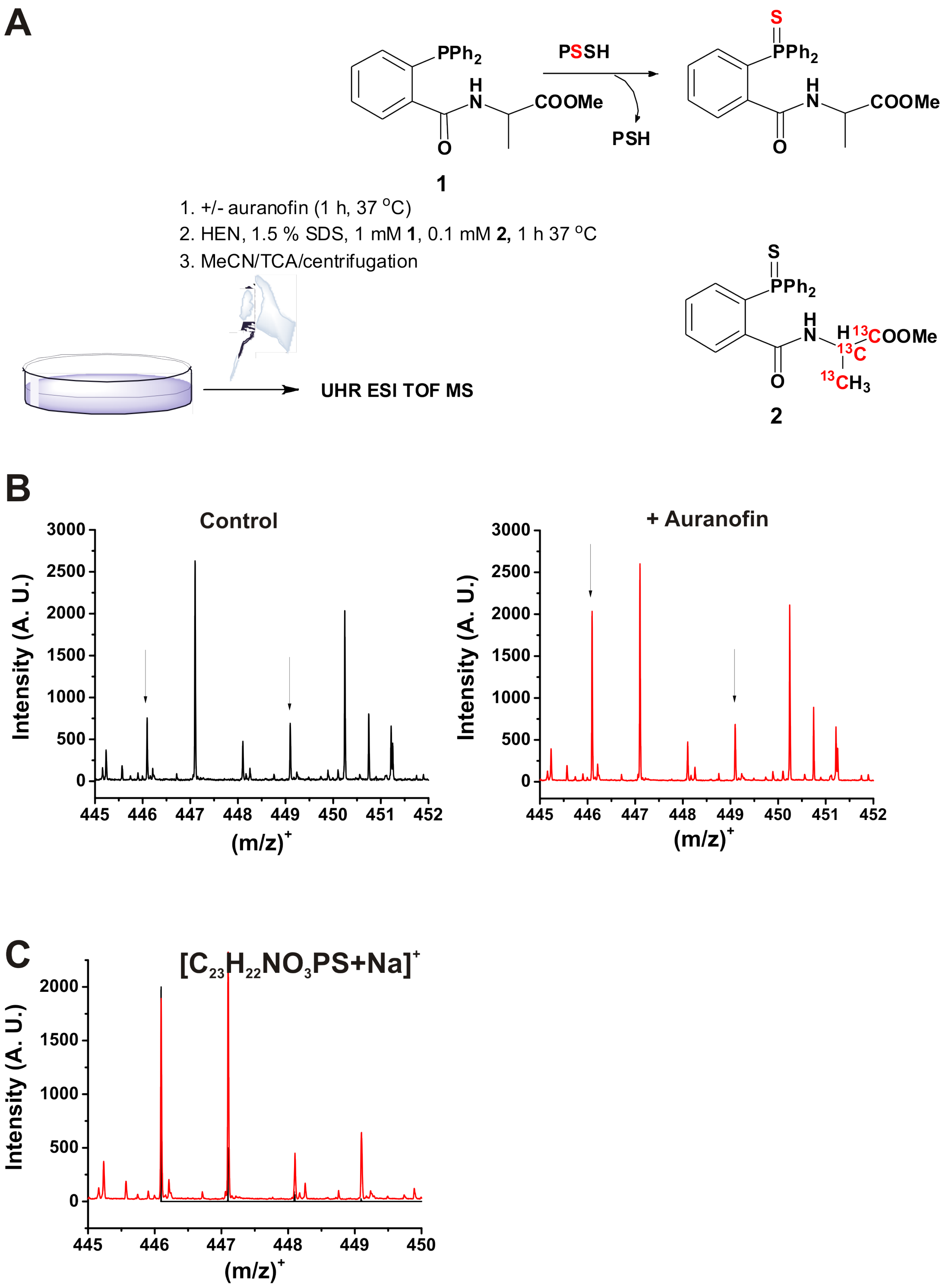

Figure S10. Isotope dilution MS method for sulfane sulphur quantification. A) Structures of probe **1** and probe **2** used for the isotope dilution method and the schematic representation of the methodology. **B)** Representative MS spectra of the control BAEC lysates (left) or of the lysates obtained from the BAECs treated with 2 μM auranofin for 1 h (right). The arrows point to the *m/z* of the detected product and isotope labelled internal standard. **C)** Observed (red) and simulated (black) isotope distribution for *m/z* 446.0956 peak corresponding to the [**1**=S + Na]$^+$.

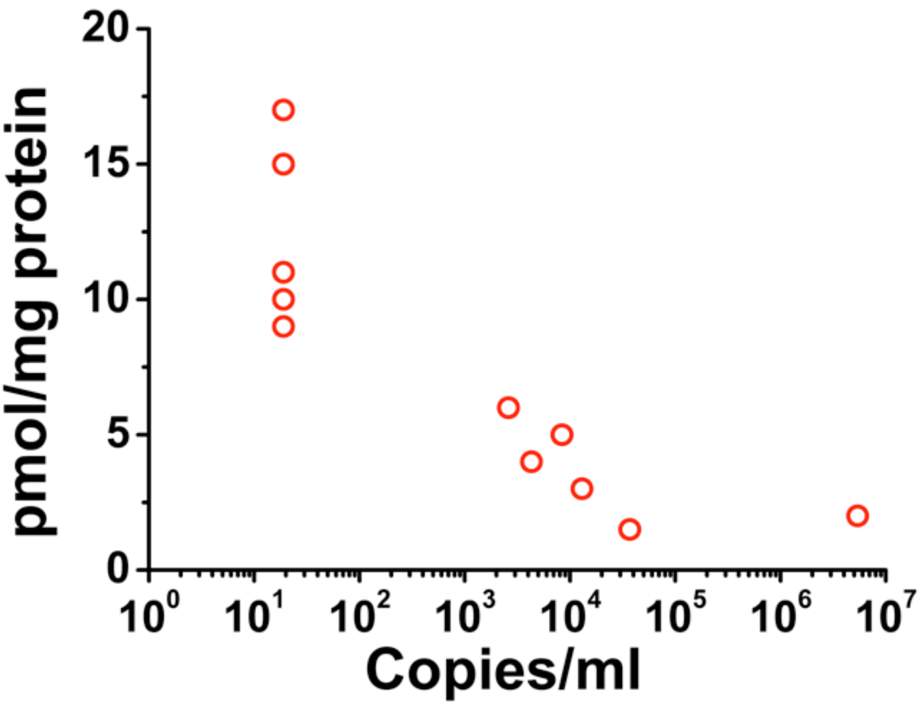

Figure S11. Sulfane sulfur levels vs. HIV-1 viral load. HIV-1 viral load is given as copies/ml. Values <20 copies/ml are indicated in the graph as 19 copies/ml. (the detection limit of the PCR is 20 copies/ml). Spearman correlation: correlation coefficient: -0.9344, p=0.000.

6-Supporting Tables

Table S1. Basic patients characteristics.

Patient number	sex	age	CDC	CD4	viral load	ART	sulfane sulphur pmol/mg
#1	f	43	B3	707	<20	yes	10
#2	m	50	A2	331	<20	yes	15
#3	m	36	B3	309	<20	yes	9
#4	m	46	C3	364	<20	yes	17
#5	f	53	C3	955	<20	yes	11
#6	m	27	A1	684	13000	no	3
#7	m	37	A1	531	8400	no	5
#8	m	29	B2	143	37000	no	1.5
#9	m	34	A3	182	2600	no	6
#10	m	42	B2	395	5400000	no	2
#11	f	39	A2	476	4300	no	4

m: male; f: female; CDC: CDC stage of HIV-1-infection; CD4: CD4+ T-cells/µl; CD8: CD8+ T-cells /µl; VL: HIV-1 viral load in plasma in copies/ml plasma; sulfane sulphur levels in pmol/mg of plasma protein; ART: antiretroviral therapy; pat#2 is treated for diabetes with metformin, sitagliptin, levemir and ASS; pat#9 suffers from chronic hepatitis C (HCV viral load 340 000 copies/ml).

7-Product Characterization

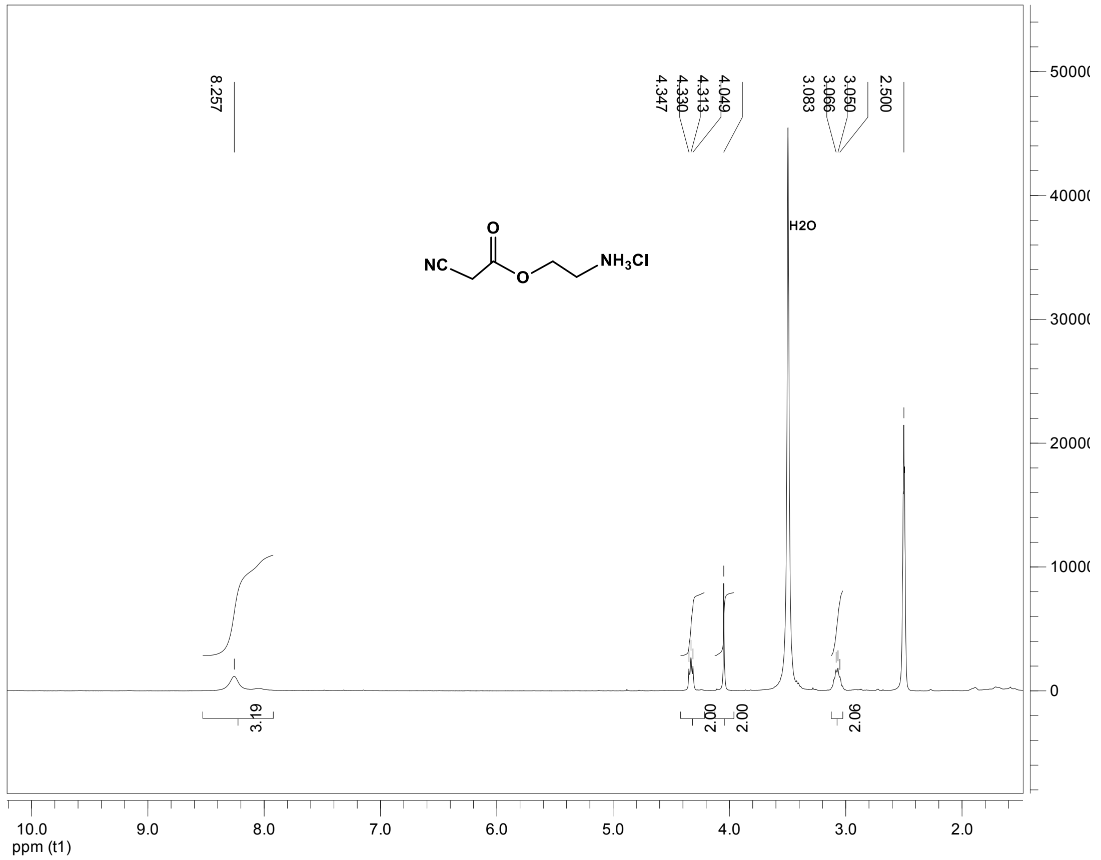

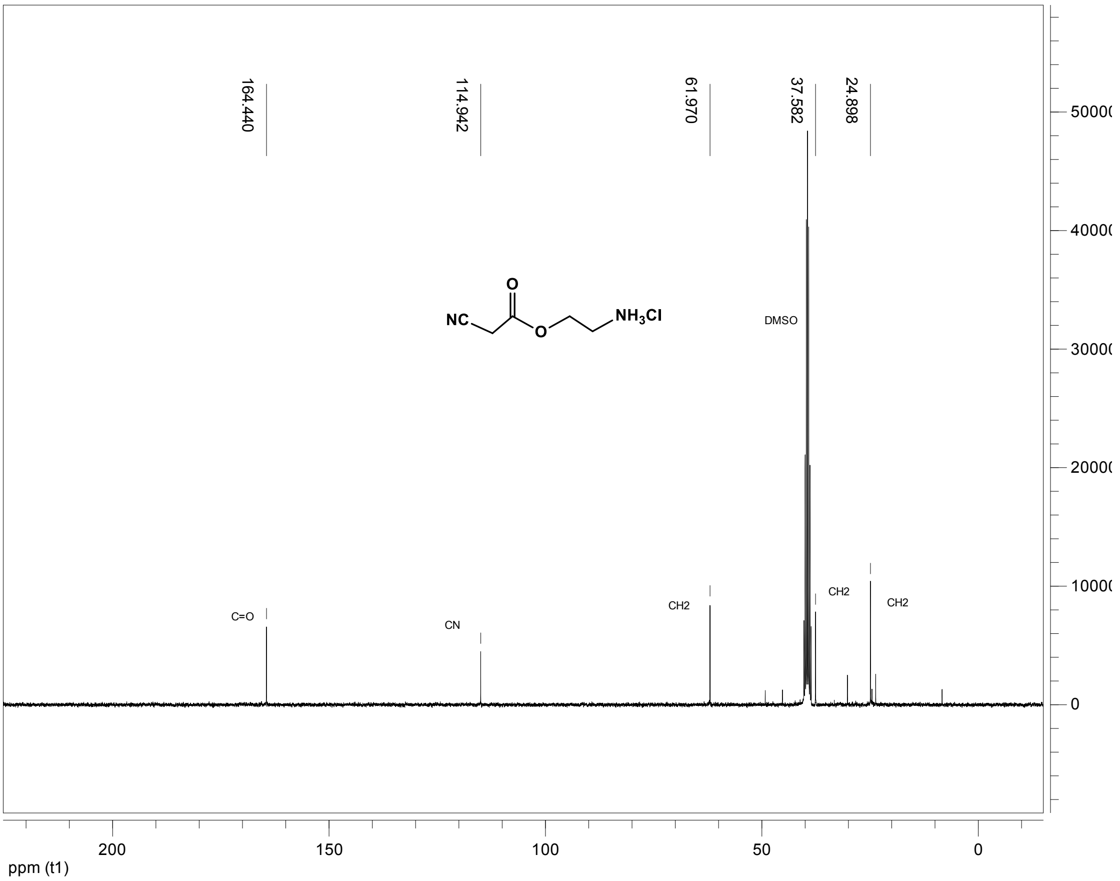

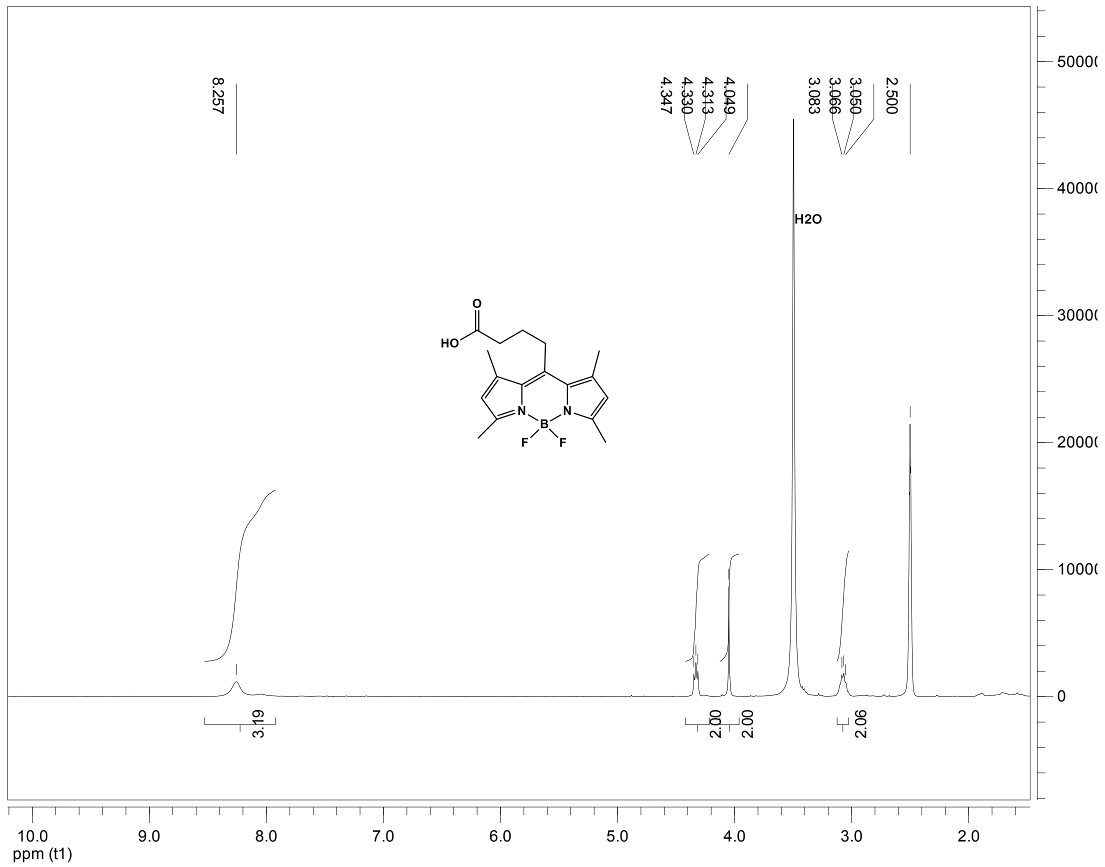

8.257
4.347
4.330
4.313
4.049
3.083
3.066
3.050
2.500
H2O
HO
O
N
B
F
3.19
2.00
2.00
2.06
10.0
9.0
8.0
7.0
6.0
5.0
4.0
3.0
2.0
ppm (t1)

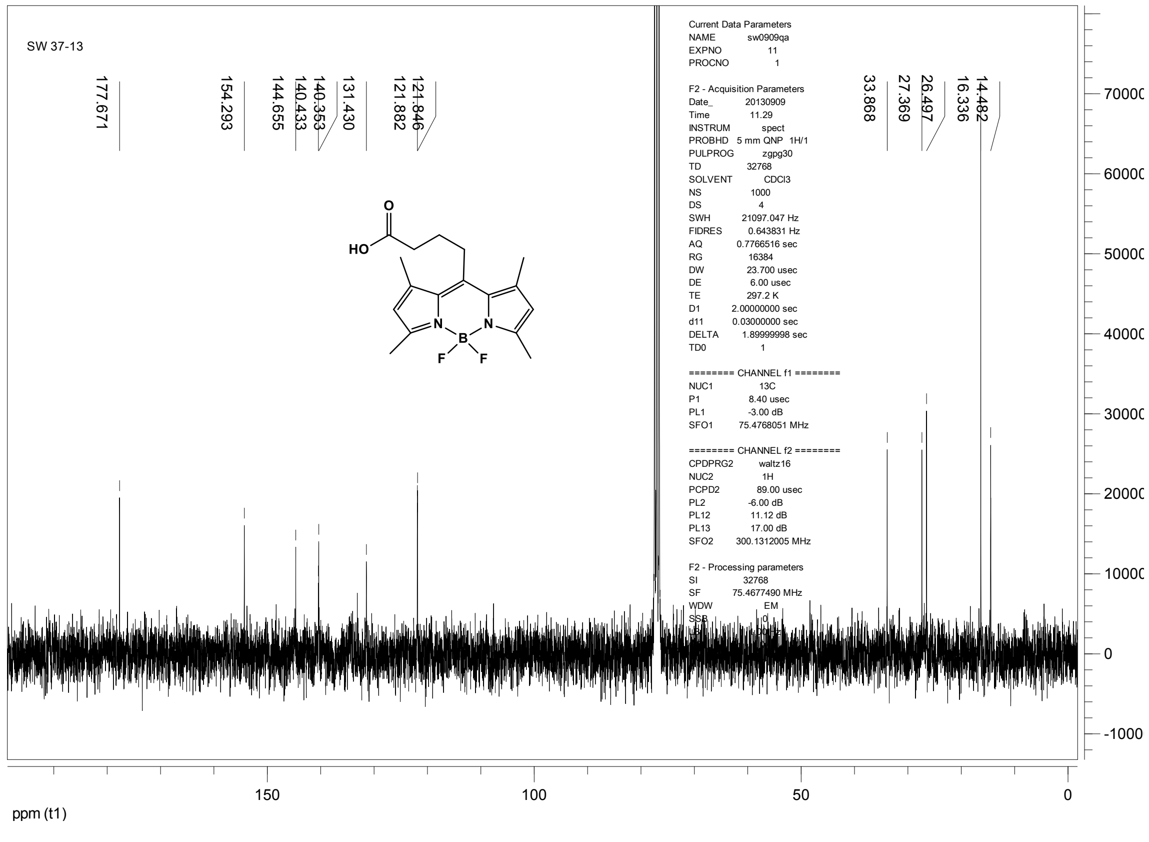

SW 37-13
177.671
154.293
144.655
140.433
140.353
131.430
121.882
121.846
33.868
27.369
26.497
16.336
14.482
Current Data Parameters
NAME sw0909qa
EXPNO 11
PROCNO 1
F2 - Acquisition Parameters
Date_ 20130909
Time 11.29
INSTRUM spect
PROBHD 5 mm QNP 1H/1
PULPROG zgpg30
TD 32768
SOLVENT CDCl3
NS 1000
DS 4
SWH 21097.047 Hz
FIDRES 0.643831 Hz
AQ 0.7766516 sec
RG 16384
DW 23.700 usec
DE 6.00 usec
TE 297.2 K
D1 2.00000000 sec
d11 0.03000000 sec
DELTA 1.89999998 sec
TD0 1
======== CHANNEL f1 ========
NUC1 13C
P1 8.40 usec
PL1 -3.00 dB
SFO1 75.4768051 MHz
======== CHANNEL f2 ========
CPDPRG2 waltz16
NUC2 1H
PCPD2 89.00 usec
PL2 -6.00 dB
PL12 11.12 dB
PL13 17.00 dB
SFO2 300.1312005 MHz
F2 - Processing parameters
SI 32768
SF 75.4677490 MHz
WDW EM
150
100
50
0
ppm (t1)

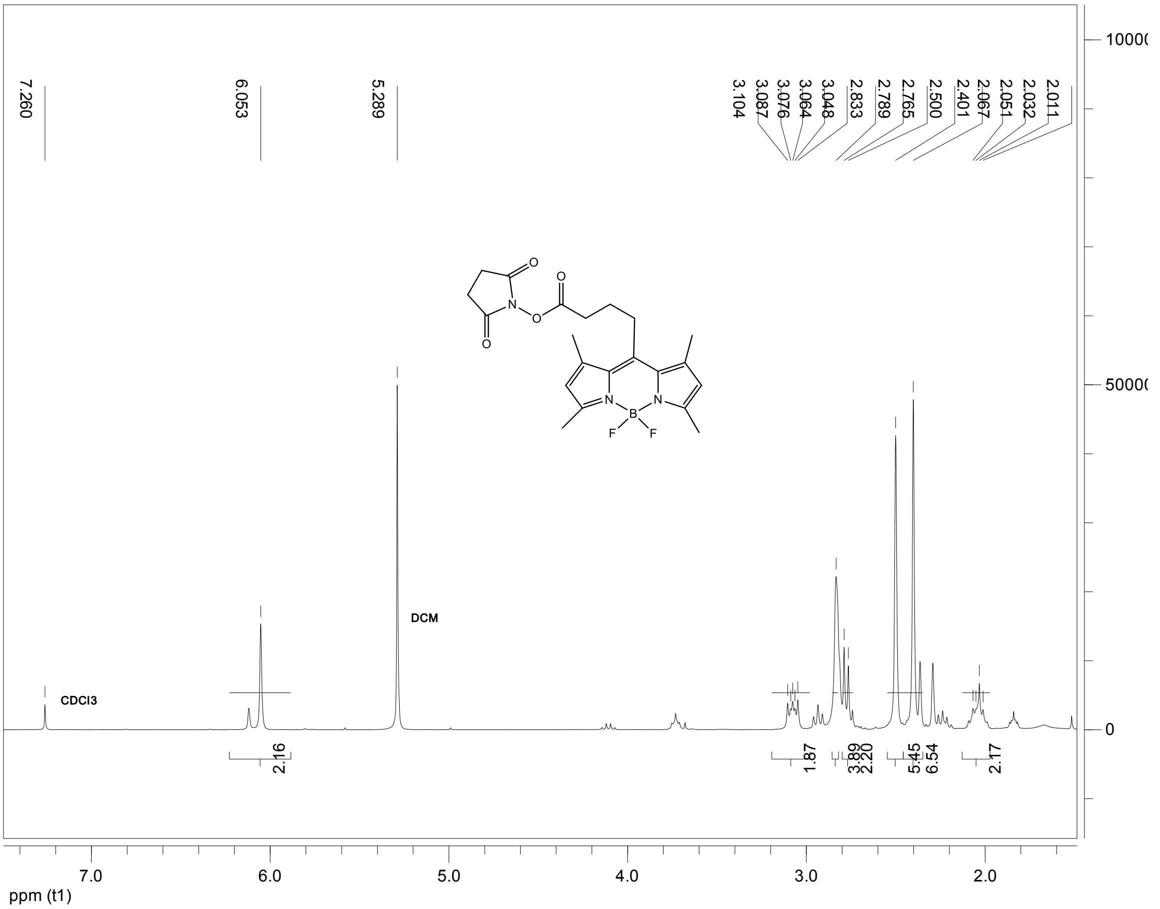
7.260
6.053
5.289
3.104
3.087
3.076
3.064
3.048
2.833
2.789
2.765
2.500
2.401
2.067
2.051
2.032
2.011
CDCl3
DCM
2.16
1.87
3.89
2.20
5.45
6.54
2.17
7.0
6.0
5.0
4.0
3.0
2.0
ppm (t1)
0
50000

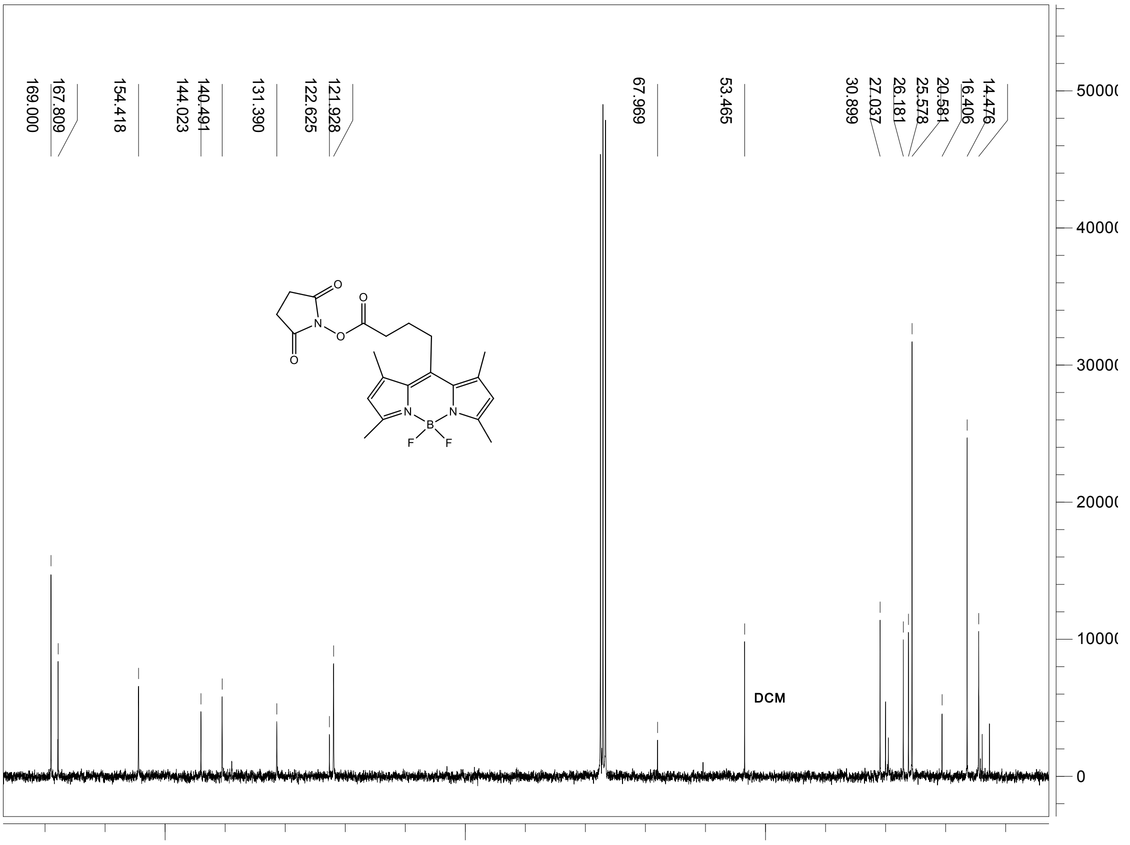
169.000
167.809
154.418
144.023
140.491
131.390
122.625
121.928
67.969
53.465
30.899
27.037
26.181
25.578
20.581
16.406
14.476
DCM
150
100
50
ppm (t1)
0
10000
20000
30000
40000
50000

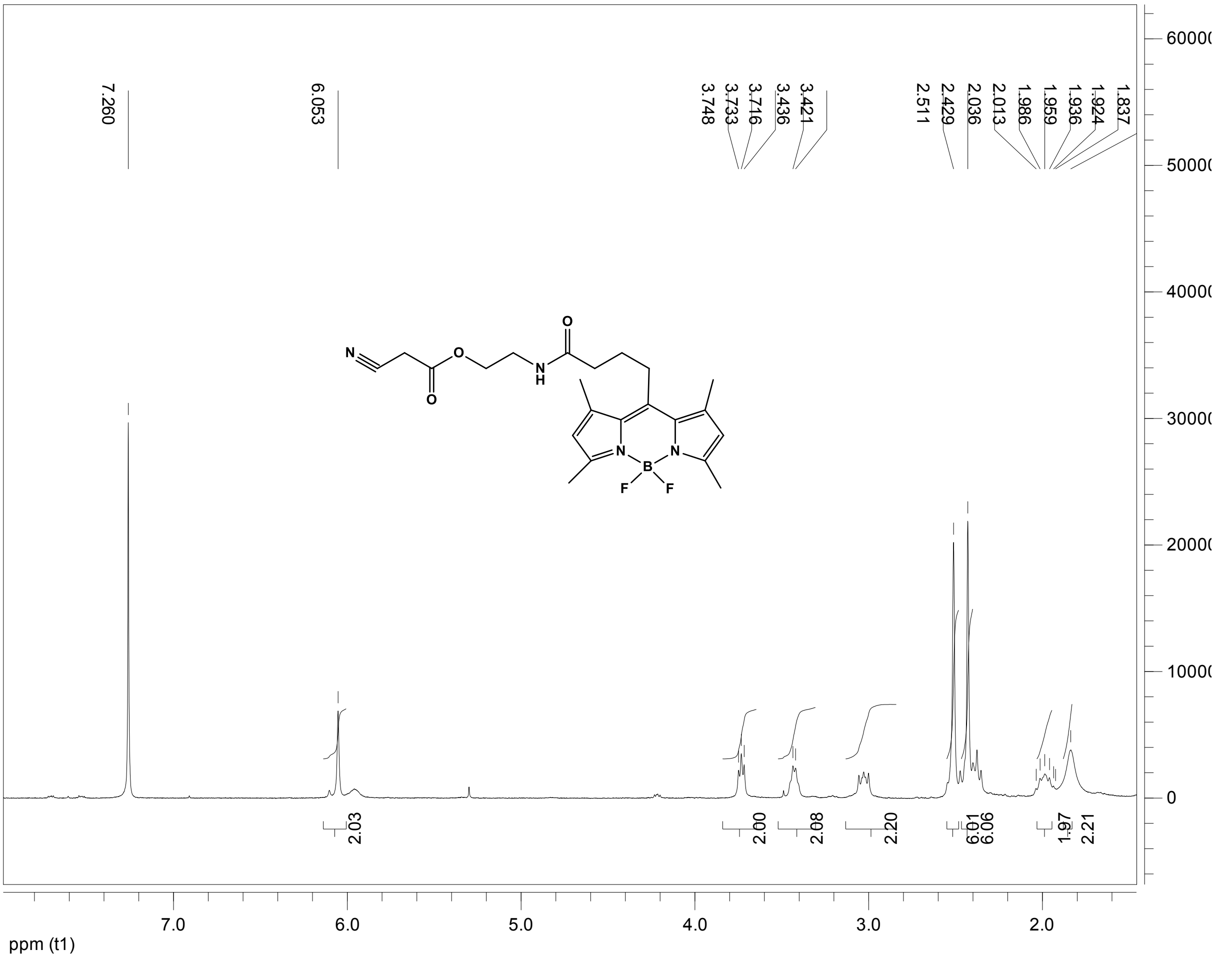

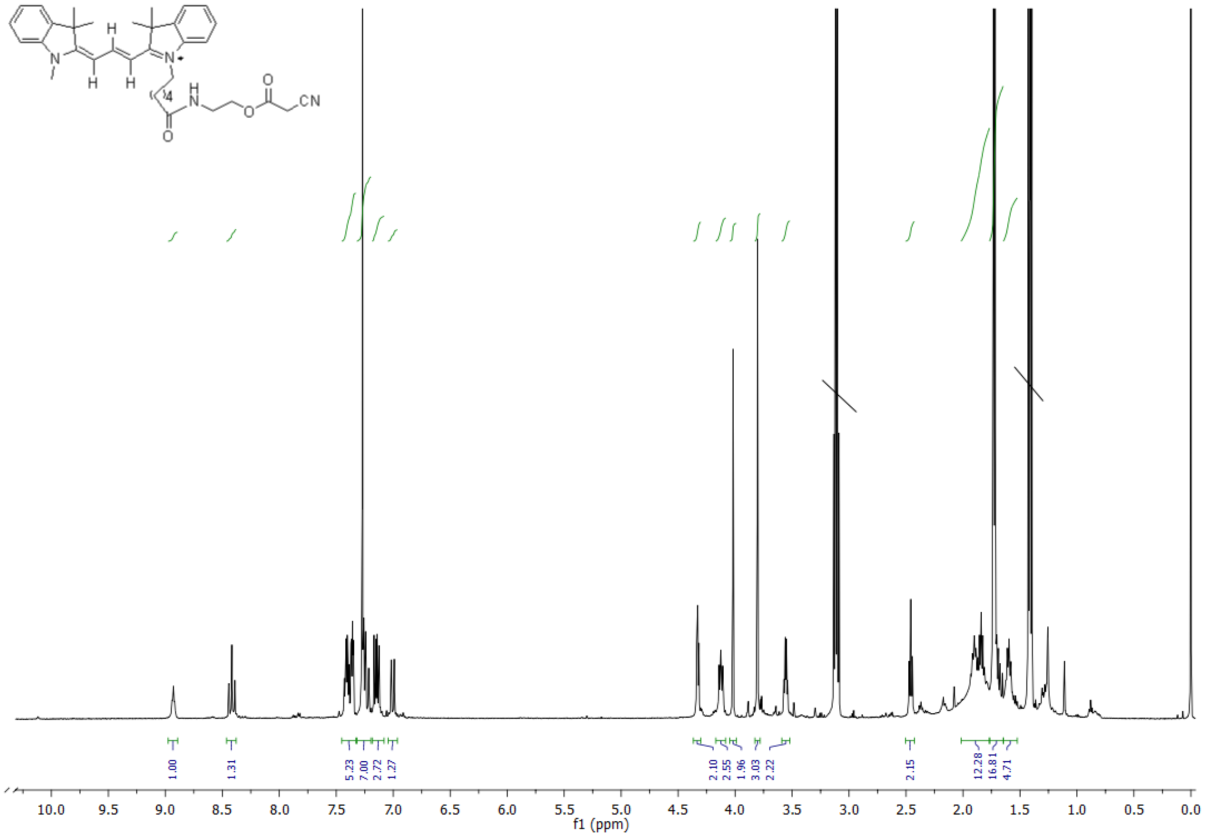

Supplement: Supplementary file 1 [file SC-007-C5SC04818D-s001.pdf]
